# Abortion decision-making trajectories and factors influencing such trajectories in low- and middle-income countries: a protocol for mixed-methods systematic review

**DOI:** 10.1136/bmjopen-2021-049507

**Published:** 2021-11-01

**Authors:** Paul Lokubal, Sandrena Ruth Frischer, Ines Corcuera, Jessica Macias Balil, Christine Nalwadda Kayemba, Jennifer J Kurinczuk, Manisha Nair

**Affiliations:** 1 National Perinatal Epidemiology Unit, Nuffield Department of Population Health, University of Oxford, Oxford, UK; 2 Partners in Health, Monrovia, Liberia; 3 Chelsea and Westminster Hospital NHS Foundation Trust, London, UK; 4 MSI Reproductive Choices UK, London, UK; 5 Community Health and Behavioural Sciences, School of Public Health, College of Health Sciences, Makerere University, Kampala, Uganda

**Keywords:** public health, health policy, maternal medicine

## Abstract

**Introduction:**

Globally, about half of all pregnancies are unintended and/or unwanted and three-fifths of these end in induced abortion. When faced with a choice to terminate pregnancy, women’s abortion decision-making processes are often complex and multiphasic and maybe amplified in low- and middle-income countries (LMICs) which bear the major burden of abortion-related morbidity and mortality. Our review aims to (1) describe abortion decision-making trajectories for women in LMICs and (2) investigate factors influencing the choice of abortion decision-making trajectories in LMICs.

**Methods and analysis:**

We will search and retrieve published and unpublished qualitative, quantitative and mixed-methods, community and/or hospital-based studies conducted in LMICs from 1 January 2000 up to 16 February 2021. We will search Ovid Medline, Ovid EMBASE, Ovid PsycInfo, Ovid Global Health, Web of Science (including Social Science Citation Index), Scopus, IBSS, CINAHL via EBSCO, WHO Global Index Medicus, the Cochrane Library, WHO website, ProQuest and Google Scholar. We will search reference lists of eligible studies and contact experts for additional data/information, if required. We will extract all relevant data to answer our research questions and assess study quality using the appropriate appraisal tools. Depending on the extracted data, our analysis will use sequential or convergent synthesis methods proposed by Hong *et al*. For qualitative studies, we will synthesise evidence using thematic synthesis, meta-ethnography or ‘best-fit’ framework synthesis; and for quantitative findings, we will provide a narrative synthesis and/or meta-analysis. We will do sensitivity analyses and assess confidence in our findings using Grades of Recommendation, Assessment, and Evaluation –Confidence in Evidence from Reviews of Qualitative Research (GRADE-CERQUal) for qualitative findings and Grades of Recommednation, Assessment, and Evaluation (GRADE) for quantitative findings.

**Ethics and dissemination:**

We did not require ethics approval for this systematic review. We will publish our findings in an open-access peer-reviewed journal with global and maternal health readership. We will also present our findings at national and international scientific conferences.

**Strengths and limitations of this study**

- The review is one of the first to synthesise evidence on abortion decision-making processes in low- andmiddle-income countries (LMICs) including abortion decision trajectories and factors influencing their choices.
- The review includes multiple databases, grey literature with no language restrictions, and covers articles published from 2000 onwards up to 16 February 2021 in order to capture the contemporary abortion decision-making process.
- The systematic review will be conducted following the Preferred Reporting Items for Systematic Reviews and Meta-Analyses guidelines; this includes the use of at least two reviewers to independently search, screen and select studies, extract data and assess quality of included studies.
- Due to the sensitivity and scarcity of studies on abortion in some LMICs, few or no studies may be available from certain countries or regions where abortion is highly restricted which may affect the generalisability of our findings and data synthesis plan.

## Introduction

Globally, an estimated 48% (121 million) of all pregnancies each year from 2015 to 2019 were unintended and/or unwanted and 61% (73 million) of these ended in induced abortion.^1 2^ The proportion of unintended and/or unwanted pregnancies that end in induced abortion is similar between low-income countries (LICs) and high-income countries (HICs) (40% and 43%, respectively) but higher in middle-income countries (MICs) (66%).^1^ Between 2010 and 2014, 45% of all abortions were estimated to be unsafe with 97% occurring in low- and middle-income countries (LMICs).^3^ The proportion of all abortions that are unsafe is about four times higher (49.5%) in LMICs compared with HICs (12.5%).^3^ The proportion of unsafe abortions is 0.9% in North America, 2.1% in Northern Europe, 37.8% in Asia, 75.6% in Africa and 76.4% in Latin America.^3^ Unsafe abortion and its complications are a major cause of avoidable maternal deaths and morbidity globally, accounting for 4.7%–13.2% of all maternal deaths,^4^ US$553 million in treatment costs in LMICs^5^ and 18 100 years lived with disability.^6^ Despite accounting for only 29% of all unsafe abortions globally, 62% of all abortion-related deaths occur in Africa.^3^

While the differences in unsafe abortion rates and related morbidity and mortality differ markedly according to a country’s gross domestic product, the overall induced abortion rates are somewhat similar worldwide.^1 2^ Globally, the highest overall abortion rates are seen in MICs and the lowest in HICs; the rates per 1000 women aged 15–49 years are 44 in MICs, 38 in LICs and 15 in HICs.^1–3^ Generally, while restrictive abortion laws make most abortions unsafe,^3^ the overall abortion rates are similar in countries with varying abortion laws.^1 2^ However, in LMICs, unsafe abortion rates are similar regardless of a country’s abortion laws.^7 8^ The majority of induced abortions are for unwanted pregnancies due to failure or non-use of contraception, rape, defilement or incest.^2^ However, even planned pregnancies can become unwanted due to changes in circumstances during pregnancy including health concerns if the pregnancy is continued to term.^2^ Other reasons for abortion include: financial concerns, parenting readiness, need to space or limit childbirth, influence from significant others (such as partners and family), lack of support for the pregnancy from partners or family members, career and education goals, and stigmatised pregnancies such as teenage or out-of-wedlock pregnancies.^9–13^

Due to the sensitivity and the socioeconomic and power dynamics involved in abortion,^14^ abortion decision-making trajectories are often complex, iterative, multiphasic, dynamic, context-specific and may involve periods of intense negotiations between the woman and the significant others.^9–12 15–19^ According to Coast *et al*, abortion decision-making trajectories are ‘the processes and transitions occurring over time for a pregnancy that ends in abortion’.^16^ The circumstances surrounding a woman’s decision to seek an abortion can be time-specific and variable.^18^ Women may ‘suffer in silence’ due to uncertainty on who to talk to about the decision to terminate a pregnancy and their reactions to such a decision.^20^ The abortion trajectories chosen may affect the safety of the abortion and access to post-abortion care.^15 20^ The particular trajectory taken is influenced by various legal, socioeconomic, demographic and cultural factors such as financial stability, relationship stability, influence of significant others, risk perceptions, stigma, knowledge of abortion laws, and availability and access to abortion services.^9 11 12 15–19^ Additionally, the increasing availability and use of misoprostol to terminate pregnancy means that women can now access abortion services outside formal healthcare systems^21^ thus bypassing legal restrictions in settings where abortion is illegal.^22^

### Rationale for the systematic review

With 97% of all unsafe abortions occurring in LMICs,^3^ it is important to synthesise evidence on the abortion decision-making processes in these settings. The aim is to conduct a systematic review to synthesise the evidence relating to abortion decision-making trajectories and their determinants in LMICs. The review will help to visualise the complex decision-making trajectories which in turn could bring to light the unrecognised factors that contribute to unsafe abortion and pave way for further research and policy actions to address unsafe abortion in LMIC settings.

### Review questions

The questions address by this systematic review are:

1. What are the abortion decision-making trajectories for women seeking abortion in LMICs?
2. For women in LMICs, what factors influence the choice of these abortion trajectories?

## Methodology

### Development of review protocol and registration

We followed the guidelines set out in the Preferred Reporting Items for Systematic Review and Meta-Analysis Protocol (PRISMA-P) 2015 statement^23^ to develop the protocol. We completed the PRISMA-P checklist (online supplemental file 1). The review protocol has been registered with the International Prospective Register of Systematic Reviews with systematic review registration number CRD42021224719.

10.1136/bmjopen-2021-049507.supp1 Supplementary data

### Searches

The search strategy will be developed with the assistance of an information librarian. The first author (PL) will search the following electronic bibliographical databases: Ovid Medline, Ovid EMBASE, Ovid PsycInfo, Ovid Global Health, Web of Science (including Social Science Citation Index), Scopus, IBSS, CINAHL via EBSCO, WHO Global Index Medicus and the Cochrane Library. PL will also search grey literature sources including ProQuest, Google Scholar and the WHO website.

References of all included articles will be checked for additional articles that may have been missed from earlier searches. In addition, we will also contact experts—with experience in the field of abortion in LMICs—for any additional articles. We will limit our search strategy to articles published from 1 January 2000. Due to time constraints, the last search date for articles will be on 16 February 2021. The year 2000 has been chosen because it marked the start of the Millennium Development Goals which included a global commitment to reduce by 75%, between 1990 and 2015, the maternal mortality ratio.^24 25^ Since then, many countries have liberalised or decriminalised abortion.^2^

There will be no language restrictions in order to maximise the relevant articles from LMICs. The search strings will be composed of the following three key concepts and their synonyms: “abortion,” “decision-making”, “developing countries” and will be written with Boolean terms. We will modify the search strings depending on database requirements and use both keywords in English and medical subject headings in the search process. We will use the search filters for LMICs from Cochrane (https://epoc.cochrane.org/lmic-filters). We will create email alerts for any new relevant articles published and rerun the searches before the final analysis to identify and retrieve any further eligible studies for inclusion. We will maintain records of all searches for each database. A sample of the search strategy from Ovid Medline that was generated by the librarian and PL is attached (online supplemental file 2).

10.1136/bmjopen-2021-049507.supp2 Supplementary data

### Eligibility criteria

#### Inclusion and exclusion criteria for studies

All eligible observational studies (cross-sectional, case–control and cohort), surveys, technical reports and intervention studies will be included in the systematic review. Although we will exclude trial registrations, systematic review protocols, systematic reviews, case series, conference abstracts, case reports, policy analyses, commentaries, conceptual frameworks and editorials from the review, we will cross-check their reference lists to identify and retrieve, if any, further articles for inclusion. We will consider all relevant published and unpublished (grey literature) quantitative, qualitative and mixed-methods studies restricted to humans.

#### Participants/population

For the studies to be included, the population studied must be women who had an induced abortion and/or other actors such as abortion care providers whether skilled or unskilled, formal or informal and women’s male partners who were directly involved in the abortion decision-making process for that induced abortion. We shall exclude studies that focus only on women with spontaneous abortions or miscarriages, or reports or opinions of healthcare providers and policymakers on abortion.

#### Intervention(s) and exposure(s)

There is no intervention for our review but our focus is to understand abortion decision-making processes in LMICs for women who undergo induced abortions. We will focus on abortion decision trajectories and factors influencing the choice of such trajectories.

#### Comparators

While having a comparator is not essential for this review, studies such as observational studies having comparison groups will not be excluded on the basis of having control or comparator groups.

#### Outcomes

The main outcomes of this review include abortion trajectories and factors influencing choices of abortion trajectories in LMICs.

#### Context or study settings

We will consider only studies conducted in LMICs as defined by World Bank^26^ irrespective of the legal status of and policy environment on abortion. We will include all relevant community and/or facility-based studies that used either primary or secondary data. We will exclude animal studies.

### Study screening and selection

We will use Covidence software to screen and select eligible studies. The study screening and selection will take place in two stages with PL involved in screening all articles from the search strategy, while second author (SRF) will screen 40% of all included articles and IC and JMB (third and fourth authors) will screen 30% each. In the first stage, the reviewers will independently screen all titles and abstracts based on inclusion criteria. All four reviewers will regularly discuss results to verify the selection process and include all relevant articles for full-text review. In the second stage, the two groups of reviewers will independently read the full texts of all selected articles and include only those mentioning either of the key outcomes including trajectories of abortion decision-making or determinants of such trajectories. For the full-text screening, the authors will resolve any disagreements by consensus or by consulting the senior author (MN) and/or the co-investigator group. We will chart the results of the screening and selection process on the PRISMA flow diagram.

### Data extraction

We will use the Covidence systematic review software to extract data and assess study quality. We will extract the following information: study aim(s); study setting (including location(s) and year(s)); inclusion/exclusion criteria and participant characteristics; study methodology (including study design, sample size, data collection and analytical methods); results (including frequencies, effect sizes, themes, quotes, author interpretations or explanations); strengths and limitations; reviewer comments and all information needed to assess the risk of bias. The extraction will be done by PL (all articles), with SRF, IC and JMB being second assessors. Two authors will extract the data independently and resolve discrepancies through discussion, involving another reviewer (MN) when necessary. We will contact authors for any missing, uncertain or incomplete information; and if there is no response within 2 weeks, we may exclude those articles based on missing information. We will first pilot our data extraction process, independently and in duplicate, on five articles and make further refinements as needed. Depending on the extracted data, we may generate single or separate data extraction templates for qualitative and quantitative findings.

### Risk of bias (quality) assessment

Each article will be assessed by two reviewers, with PL reviewing all articles and SRF, IC and JMB being the second assessors. We anticipate that the majority of studies will be qualitative with few or no quantitative observational studies and experimental studies. We will use the most appropriate quality assessment tools for the studies included.^27^ The assessment will therefore be based on the articles included and will involve at least two reviewers assessing each article independently.

We will use the revised 2019 version of the Cochrane risk of bias tool^28^ to assess randomised controlled trials (RCTs) if we find any. To assess the quality of non-RCTs, we will use the Risk Of Bias In Non-randomised Studies–of Interventions.^29^ We will rate the overall quality assessment as low, moderate, serious, critical or no information provided.^29^

For cohort and case–control studies, we will use the Newcastle–Ottawa Scale.^30 31^ This tool is best for cohort and case–control studies as it allows user modification.^27^ For analytical or descriptive cross-sectional studies, we will use the Joanna Briggs Institute assessment tool.^32 33^

For qualitative studies, we will use the critical appraisal skills programme appraisal checklist for qualitative studies and assign each paper an overall quality ranking of ‘low’, ‘medium’ or ‘high’.^34^

Each reviewer will independently assess and rate each included study using the relevant quality assessment tool. We will discuss the quality assessment and risk of bias assessment findings and resolve any disagreements by consensus or by involving the senior author (MN) if necessary. For ‘poor’ quality qualitative studies, we will contact the authors for more information, a standard practice for assessing quality of qualitative studies.^35^ We will not exclude any studies based on quality assessment.^36^ We will present results of quality assessment in tabular form with comments or explanations.

### Strategy for data synthesis

While no widely accepted approach is available for synthesising a mixed-methods systematic review, and any approach chosen depends on the type of studies (qualitative, quantitative or mixed-methods) and the purpose of the research,^37^ we will analyse the data on the basis of the findings from our search. We anticipate that there will be mainly qualitative studies and the quantitative studies available may not be sufficient for meta-analysis or the findings are likely to be heterogeneous. If this is the case, we will provide a narrative summary of the quantitative findings. However, if there are sufficient quantitative studies, we will follow one of the two approaches in the synthesis as suggested by Hong *et al*^38^: (1) sequential synthesis design involving two phases—in phase one, we will first identify the main themes or components of the research questions using qualitative synthesis; in phase two, we will analyse quantitative studies to quantify the effect of each component or theme; or (2) convergent synthesis design—we will analyse qualitative and quantitative studies separately and integrate the findings at the results or discussion stage. We will use the results to develop an abortion decision-making model for women in LMICs from our analysis.

For qualitative analysis, we will upload extracted information into NVivo software to support the qualitative analysis. We will follow the thematic analysis approach developed by Thomas and Harden in 2008 to synthesise the qualitative data.^39^ The analytical approach has three stages namely: (1) developing coding schemes, (2) developing descriptive themes from the coding schemes and (3) generating analytical themes from the descriptive themes.^39^ However, depending on the extracted data, we may follow other approaches such as meta-ethnography^40 41^ or ‘best-fit’ framework synthesis^42 43^ using the trajectories of women’s abortion-related care conceptual framework developed by Coast *et al*^16^ as a template. We will add other domains and subdomains or modify existing ones, depending on the data we extract.

For the quantitative synthesis, we will extract the quantitative data into an Excel sheet and then export these to the statistical software package Stata. For categorical variables, we will analyse pooled estimates using a random-effects model. For continuous variables, we will calculate a pooled difference of means with 95% CIs using a DerSimonian-Laird random-effects model. If the mean and SD are not reported or are unavailable from the study authors, we will estimate them from sample size, median, range and/or IQR using the methods described by Wan *et al*.^44^ If we identify sufficient studies, we will conduct subgroup analysis by countries’ abortion laws, World Bank economic group and geographical regions. We will also conduct a sensitivity analysis excluding studies with low quality. We will assess heterogeneity using the I^2^ test and publication bias using forest plots. We will only assess for publication bias if there are at least 10 studies included in the meta-analysis.^45^

Following recommendations from the Cochrane Qualitative and Implementation Methods Group,^46^ we will report external validity of key qualitative synthesis using the Grades of Recommendation, Assessment, and Evaluation –Confidence in Evidence from Reviews of Qualitative Research (GRADE-CERQUal).^47^ We will use the Grades of Recommendation, Assessment, and Evaluation (GRADE) guidelines to assess the quality of any quantitative findings.^48^

### Patient and public involvement

The data from the systematic review include previously published data and will therefore not involve any patients or the public.

### Ethics and dissemination

We did not require ethics approval for this systematic review. We will publish our findings in an open-access peer-reviewed journal with global health and maternal health readership. We will also present our findings at national and international scientific conferences.

## Supplementary Material

Author's manuscript
