# Supplementary material for: Abortion decision-making trajectories and factors influencing such trajectories in low- and middle-income countries: a protocol for mixed-methods systematic review
Source: BMJ Open. 2021 Nov 1;11(11):e049507. doi: 10.1136/bmjopen-2021-049507 (PMC8562532; doi:10.1136/bmjopen-2021-049507)
Supplement: Author's manuscript [file bmjopen-2021-049507.draft_revisions.pdf]

BMJ Open is committed to open peer review. As part of this commitment we make the peer review history of every article we publish publicly available.

When an article is published we post the peer reviewers' comments and the authors' responses online. We also post the versions of the paper that were used during peer review. These are the versions that the peer review comments apply to.

The versions of the paper that follow are the versions that were submitted during the peer review process. They are not the versions of record or the final published versions. They should not be cited or distributed as the published version of this manuscript.

BMJ Open is an open access journal and the full, final, typeset and author-corrected version of record of the manuscript is available on our site with no access controls, subscription charges or pay-per-view fees (http://bmjopen.bmj.com).

If you have any questions on BMJ Open's open peer review process please email info.bmjopen@bmj.com

BMJ Open

Abortion decision-making trajectories and factors influencing such trajectories in low- and middle-income countries: a protocol for mixed methods systematic review

Journal:	*BMJ Open*
Manuscript ID	bmjopen-2021-049507
Article Type:	Protocol
Date Submitted by the Author:	26-Jan-2021
Complete List of Authors:	Lokubal, Paul; University of Oxford, Nuffield Department of Population Health (NDPH)<br>Frischer, Sandrena Ruth; Partners in Health, Monrovia<br>Corcuera, Ines; Chelsea and Westminster Hospital NHS Foundation Trust<br>Balil, Jessica ; MSI Reproductive Choices UK<br>Nalwadda Kayemba, Christine ; Karolinska Institute/Makerere University<br>Kurinczuk, Jennifer; University of Oxford, National Perinatal Epidemiology Unit<br>Nair, Manisha; University of Oxford, NPEU, Nuffield Department of Population Health
Keywords:	PUBLIC HEALTH, Health policy < HEALTH SERVICES ADMINISTRATION & MANAGEMENT, Maternal medicine < OBSTETRICS

SCHOLARONE™
Manuscripts

I, the Submitting Author has the right to grant and does grant on behalf of all authors of the Work (as defined in the below author licence), an exclusive licence and/or a non-exclusive licence for contributions from authors who are: i) UK Crown employees; ii) where BMJ has agreed a CC-BY licence shall apply, and/or iii) in accordance with the terms applicable for US Federal Government officers or employees acting as part of their official duties; on a worldwide, perpetual, irrevocable, royalty-free basis to BMJ Publishing Group Ltd ("BMJ") its licensees and where the relevant Journal is co-owned by BMJ to the co-owners of the Journal, to publish the Work in this journal and any other BMJ products and to exploit all rights, as set out in our licence.

The Submitting Author accepts and understands that any supply made under these terms is made by BMJ to the Submitting Author unless you are acting as an employee on behalf of your employer or a postgraduate student of an affiliated institution which is paying any applicable article publishing charge ("APC") for Open Access articles. Where the Submitting Author wishes to make the Work available on an Open Access basis (and intends to pay the relevant APC), the terms of reuse of such Open Access shall be governed by a Creative Commons licence – details of these licences and which Creative Commons licence will apply to this Work are set out in our licence referred to above.

Other than as permitted in any relevant BMJ Author's Self Archiving Policies, I confirm this Work has not been accepted for publication elsewhere, is not being considered for publication elsewhere and does not duplicate material already published. I confirm all authors consent to publication of this Work and authorise the granting of this licence.

For peer review only

Abortion decision-making trajectories and factors influencing such trajectories in low- and middle-income countries: a protocol for mixed methods systematic review.

Co-authors:

1. Paul Lokubal, MSc; National Perinatal Epidemiology Unit (NPEU), Nuffield Department of Population Health (NDPH), University of Oxford, Old Road Campus, Headington, Oxford OX3 7LF, Oxford, UK
2. Sandrena Ruth Frischer, MSc; Partners in Health, Monrovia, Liberia
3. Ines Corcuera, MSc; Chelsea and Westminster Hospital, NHS Foundation Trust, 369 Fulham Road, Chelsea, London SW10 9NH, London, UK
4. Jessica Macias Balil, MSc; MSI Reproductive Choices, Conway St, Fitzroy Square, Fitzrovia, London W1T 6LP, London, UK
5. Christine Nalwadda Kayemba, PhD; Department of Community Health and Behavioural Sciences, School of Public, Makerere University College of Health Sciences, Makerere University, Kampala, Uganda
6. Jennifer J Kurinczuk, MD; National Perinatal Epidemiology Unit (NPEU), Nuffield Department of Population Health (NDPH), University of Oxford, Old Road Campus, Headington, Oxford OX3 7LF, Oxford, UK
7. Manisha Nair, DPhil; National Perinatal Epidemiology Unit (NPEU), Nuffield Department of Population Health (NDPH), University of Oxford, Old Road Campus, Headington, Oxford OX3 7LF, Oxford, UK

Corresponding author: Paul Lokubal, DPhil Student; National Perinatal Epidemiology Unit (NPEU), Nuffield Department of Population Health (NDPH), University of Oxford, Old Road Campus, Headington, Oxford OX3 7LF, Oxford, UK. paul.lokubal@ndph.ox.ac.uk

Systematic Registration: PROSPERO 2021: CRD42021224719

Key words: abortion, decision-making, abortion trajectories, low- and middle-income countries, mixed methods, systematic review, meta-analysis.

For peer review only - http://bmjopen.bmj.com/site/about/guidelines.xhtml

ABSTRACT

Introduction: Globally, about half of all pregnancies are unintended and three-fifths of these end in induced abortion. When faced with a choice to terminate pregnancy, women's abortion decision-making processes are often complex and multiphasic and maybe amplified in low- and middle-income countries (LMICs) which bear the major burden of abortion-related morbidity and mortality. Our review aims to 1) describe trajectories for women seeking abortion and post abortion care in LMICs and 2) investigate factors influencing the choice of the abortion trajectories that women in LMICs make.

Methodology: We will search and retrieve published and unpublished qualitative, quantitative and mixed methods, community or hospital-based studies conducted in LMICs from 1st January 2000. We will search Ovid Medline, Ovid EMBASE, Ovid PsychInfo, Ovid Global Health, Web of Science (including Social Science Citation Index), Scopus, IBSS, CINAHL via EBSCO, WHO Global Index Medicus, the Cochrane Library, WHO website, ProQuest, and Google Scholar. We will search reference lists of eligible studies and contact experts for additional data/ information, if required. We will extract all relevant data to answer our research questions and assess study quality using the appropriate appraisal tools. Depending on the extracted data, our analysis will use sequential or convergent synthesis methods proposed by Hong *et al*. For qualitative studies, we will synthesise evidence using thematic synthesis, meta-ethnography or "best-fit" framework synthesis and for quantitative findings, we will do descriptive synthesis and meta-analysis. We will do sensitivity analyses and assess confidence in our findings using GRADE-CERQual for qualitative findings and GRADE for quantitative findings.

Discussion: The findings of this systematic review will improve our understanding of the decision-making processes including trajectories and determinants for seeking abortion and post-abortion care in LMICs.

For peer review only - http://bmjopen.bmj.com/site/about/guidelines.xhtml

Strengths and limitations of this study

- The review is one of the first to synthesize evidence on abortion decision-making processes in LMICs including abortion decision trajectories and factors influencing their choices.
- The review includes multiple databases, grey literature with no language restrictions and covers articles published from 2000 onwards in order to capture the contemporary abortion decision-making process.
- The systematic review will be conducted following the PRISMA guidelines, this includes the use of at least two reviewers to independently search, screen and select, extract data, and assess quality of included studies.
- Due to the sensitivity and scarcity of studies on abortion in some LMICs, few or no studies may be available from certain countries or regions where abortion is highly restricted which may affect our results and data synthesis plan.

INTRODUCTION

Globally, an estimated 48% (121 million) of all pregnancies each year from 2015-2019 were unintended and 61% (73 million) of these ended in induced abortion [1, 2]. The proportion of unintended pregnancies that end in induced abortion is similar between low-income countries (LICs) and high- income countries (HICs) (40% and 43% respectively) but higher in middle-income countries (MICs) (66)% [1]. Between 2010 and 2014, 45% of all abortions were estimated to be unsafe with 97% occurring in low- and middle- income countries (LMICs) [3]. The proportion of all abortions that are unsafe is about four times higher (49.5%) in LMICs compared to HICs (12.5%) [3]. The proportion of unsafe abortions is 0.9% in North America, 2.1% in Northern Europe, 37.8% in Asia, 75.6% in Africa, and 76.4% in Latin America [3]. Unsafe abortion and its complications are a major cause of avoidable maternal deaths and

For peer review only - http://bmjopen.bmj.com/site/about/guidelines.xhtml

morbidity globally, accounting for 4.7-13.2% of all maternal deaths [4], USD 553 million in treatment costs in LMICs [5], and 18,100 years lived with disability (YLDs) [6]. Despite accounting for only 29% of all unsafe abortions globally, 62% of all abortion-related deaths occur in Africa [3].

While the differences in unsafe abortion rates and related morbidity and mortality differ markedly according to a country's Gross Domestic Product (GDP), the overall induced abortion rates are somewhat similar worldwide [1, 2]. Globally, the highest overall abortion rates are seen in MICs and the lowest in HICs; the rates per 1,000 women aged 15-49 are 44 in MICs, 38 in LICs and 15 in HICs [1–3]. Generally, while restrictive abortion laws make most abortions unsafe [3], the overall abortion rates are similar in countries with varying abortion laws [1, 2]. However, in LMICs unsafe abortion rates are similar regardless of a country's abortion laws [7, 8]. The majority of induced abortions are for unwanted pregnancies due to failure or non-use of contraception, rape, defilement, or incest [2]. However, even planned pregnancies can become unwanted due to changes in circumstances during pregnancy including health concerns if the pregnancy is continued to term [2]. Other reasons for abortion include: financial concerns, parenting readiness, need to space or limit childbirths, influence from significant others (such as partners and family), lack of support for the pregnancy from partners or family members, career and education goals, and stigmatised pregnancies such as teenage or out of wedlock pregnancies [9–13].

Due to the sensitivity and the socioeconomic and power dynamics involved in abortion [14], abortion decision-making trajectories are often complex, iterative, multiphasic, dynamic, context-specific and may involve periods of intense negotiations between the woman and the significant others [9–12, 15–19]. According to Coast *et al.*, abortion decision-making trajectories are "*the processes and transitions occurring over time for a pregnancy that ends in abortion"* [16]. The circumstances surrounding a woman's decision to seek an abortion can be time-specific and

For peer review only - http://bmjopen.bmj.com/site/about/guidelines.xhtml

variable [18]. Women may "suffer in silence" due to uncertainty on who to talk to about the decision to terminate a pregnancy and their reactions to such a decision [20]. The abortion trajectories chosen may affect the safety of the abortion and access to post-abortion care [15, 20]. The particular trajectory taken is influenced by various legal, socioeconomic, demographic, and cultural factors such as financial stability, relationship stability, influence of significant others, risk perceptions, stigma, knowledge of abortion laws, and availability and access to abortion services [9, 11, 12, 15–19]. Additionally, the increasing availability and use of misoprostol to terminate pregnancy means that women can now access abortion services outside formal health care systems [21].

Rationale for the systematic review

With 97% of all unsafe pregnancies occurring in LMICs [3], it is important to synthesise evidence on the abortion decision-making processes in these settings. The aim is to conduct a systematic review to synthesize the evidence relating to abortion decision trajectories and factors that influence those trajectories in LMICs. This will be used to develop a decision-making model or framework, for use by governments, policy makers, programme managers, researchers, and other relevant stakeholders to design and implement strategies to reduce unsafe abortion and its complications.

Review questions

The questions address by this systematic review are:

1. What are the trajectories for women seeking abortion in LMICs?
2. For women in LMICs, what factors influence the choice of these abortion trajectories?

METHODOLOGY

For peer review only - http://bmjopen.bmj.com/site/about/guidelines.xhtml

Development of review protocol and registration

We followed the guidelines set out in the preferred reporting items for systematic review and meta-analysis protocol (PRISMA-P) 2015 statement [22] to develop the protocol. We completed the PRISMA-P checklist (supplementary file 1). The review protocol has been registered with international prospective register of systematic reviews (PROSPERO) with systematic review registration number CRD42021224719.

Searches

The search strategy will be developed with the assistance of an information librarian. PL will search the following electronic bibliographic databases: Ovid Medline, Ovid EMBASE, Ovid PsychInfo, Ovid Global Health, Web of Science (including Social Science Citation Index), Scopus, IBSS, CINAHL via EBSCO, WHO Global Index Medicus, and the Cochrane Library. PL will also search grey literature sources including ProQuest, Google Scholar and the WHO website.

All references of all included articles will be checked for additional articles that may have been missed from earlier searches. In addition, we will also contact experts in the field for any additional articles. We will limit our search strategy to articles published from January 1st, 2000. The year 2000 has been chosen because it marked the start of the Millennium Development Goals (MDGs) which included a global commitment to reduce by 75%, between 1990 and 2015, the maternal mortality ratio [23, 24]. Since then, many countries have liberalised abortion laws or decriminalised abortion [2].

There will be no language restrictions in order to maximise the relevant articles from LMICs. The search strings will be composed of the following three key concepts and their synonyms: “abortion,” “decision-making”, “developing countries” and will be written with Boolean terms. We

For peer review only - http://bmjopen.bmj.com/site/about/guidelines.xhtml
For peer review only

will modify the search strings depending on database requirements and use both key words and medical subject headings (MeSH) in the search process. We will use the search filters for LMICs from Cochrane (https://epoc.cochrane.org/lmic-filters). We will create email alerts for any new relevant articles published and re-run the searches before the final analysis to identify and retrieve any further eligible studies for inclusion. We will maintain records of all searches for each database. A sample of the search strategy from Ovid Medline that was generated by the Librarian and PL is attached (supplementary file 2).

Eligibility Criteria

Inclusion and exclusion criteria for studies

All eligible observational studies (cross-sectional, case-control, and cohort), surveys, technical reports, and intervention studies will be included in the systematic review. Although we will exclude trial registrations, systematic review protocols, systematic reviews, case series, conference abstracts, case reports, policy analyses, commentaries, conceptual frameworks, and editorials from the review, we will cross-check their reference lists to identify and retrieve, if any, further articles for inclusion. We will consider all relevant published and unpublished (grey literature) quantitative, qualitative, and mixed methods studies restricted to humans.

Participants/Population

For the studies to be included, the population studied must be women who had an induced abortion and/or other actors such as abortion care providers whether skilled or unskilled, formal or informal and women's male partners who were directly involved in the abortion decision-making process for that induced abortion. We shall exclude studies that focus only on women with spontaneous abortions or miscarriages, or reports or opinions of health care providers, policy makers, or male partners on abortion.

For peer review only - http://bmjopen.bmj.com/site/about/guidelines.xhtml

Intervention(s), exposure(s)

There is no intervention for our review but our focus is to understand abortion decision-making processes in LMICs in women who are faced with a decision to terminate a pregnancy. We will focus on abortion decision trajectories and factors influencing the choice of such trajectories.

Comparators

While having a comparator is not essential for this review, studies such as observational studies having comparison groups will not be excluded on the basis of having control or comparator groups.

Outcomes

The main outcomes of this review include abortion trajectories and factors influencing choices of abortion trajectories in LMICs.

Context or study settings

We will consider only studies conducted in LMICs as defined by World Bank [25] irrespective of the legal status of and policy environment on abortion. We will include all relevant community or facility-based studies that used either primary or secondary data. We will exclude animal studies.

Study screening and selection

We will use Covidence software to screen and select eligible studies. The study screening and selection will take place in two stages with PL involved in screening all articles from the search strategy while SF will screen 40% of all included articles and IC and JM will screen 30% each. In the first stage, the reviewers will independently screen all titles and abstracts based on

For peer review only - http://bmjopen.bmj.com/site/about/guidelines.xhtml

inclusion criteria. All four reviewers will regularly discuss results to verify the selection process and include all relevant articles for full text-review. In the second stage, the two groups of reviewers will independently read the full texts of all selected articles and include only those mentioning either of the key outcomes including trajectories of abortion decision-making or determinants of such trajectories. For the full-text screening, the authors will resolve any disagreements by consensus or by consulting the senior author (MN) and/or the coinvestigator group. We will chart the results of the screening and selection process on the PRISMA flow diagram.

Data extraction

We will use the Covidence systematic review software to extract data and assess study quality. We will extract the following information: study aim(s); study setting (including location(s) and year(s)); inclusion/exclusion criteria and participant characteristics; study methodology (including study design, sample size, data collection and analytical methods); results (including frequencies, effect sizes, themes, quotes, author interpretations or explanations); strengths and limitations; reviewer comments; and all information needed to assess the risk of bias. The extraction will be done by PL (all articles), with SF, IC and JM being second assessors. Two authors will extract the data independently and resolve discrepancies through discussion, involving another reviewer (MN) when necessary. We will contact authors for any missing, uncertain, or incomplete information and if there is no response within 2 weeks, we may exclude those articles based on missing information. We will first pilot our data extraction process, independently and in duplicate, on five articles and make further refinements as needed. Depending upon the extracted data, we may generate single or separate data extraction templates for qualitative and quantitative findings.

Risk of bias (quality) assessment

For peer review only - http://bmjopen.bmj.com/site/about/guidelines.xhtml

Each article will be assessed by two reviewers, with PL reviewing all articles and SF, IC, and JM being the second assessors. We anticipate that the majority of studies will be qualitative with few or no observational studies and experimental studies. We will use the most appropriate quality assessment tools for the studies included [26]. The assessment will therefore be based on the articles included and will involve at least two reviewers assessing each article independently.

We will use the revised 2019 version of the Cochrane risk of bias tool (RoB 2) [27] to assess randomised controlled trials (RCTs) if we find any. To assess the quality of non-randomised controlled trials (non-RCTs), we will use the Risk Of Bias In Non-randomised Studies – of Interventions (ROBINS-I) [28]. We will rate the overall quality assessment as low, moderate, serious, critical or no information provided [28].

For cohort and case-control studies, we will use the Newcastle-Ottawa Scale (NOS) [29, 30]. This tool is best for cohort and case control studies as it allows user modification [26]. For analytical or descriptive cross-sectional studies, we will use the Joanna Briggs Institute (JBI) assessment tool [31, 32].

For qualitative studies, we will use the critical appraisal skills programme (CASP) appraisal checklist for qualitative studies and assign each paper an overall quality ranking of “low,” “medium,” or “high” [33].

Each reviewer will independently assess and rate each included study using the relevant quality assessment tool. We will discuss the quality assessment and risk of bias assessment findings and resolve any disagreements by consensus or by involving the senior author (MN) if necessary. For “poor” quality qualitative studies, we will contact the authors for more information, a standard practice for assessing quality of qualitative studies [34]. We will not

For peer review only - http://bmjopen.bmj.com/site/about/guidelines.xhtml

exclude any studies based on quality assessment [35]. We will present results of quality assessment in tabular form with comments or explanations.

Strategy for data synthesis

While no widely accepted approach is available for synthesising a mixed methods systematic review, and any approach chosen depends upon the type of studies (qualitative, quantitative or mixed methods) and the purpose of the research [36], we will analyse the data on the basis of the findings from our search. We anticipate that there will be mainly qualitative studies and the quantitative studies available may not be sufficient for meta-analysis or the findings are likely to be heterogeneous. If this is the case, we will provide a narrative summary of the quantitative findings. However, if there are sufficient quantitative studies, we will follow one of the two approaches in the synthesis as suggested by Hong *et al.* [37]: (1) sequential synthesis design involving two phases: in phase one, we will first identify the main themes or components of the research questions using qualitative synthesis. In phase two, we will analyse quantitative studies to quantify the effect of each component or theme; or (2) convergent synthesis design – we will analyse qualitative and quantitative studies separately and integrate the findings at the results or discussion stage. We will use the results to develop an abortion decision-making model for women in LMICs from our analysis.

For qualitative analysis, we will upload extracted information into NVIvo software to support the qualitative analysis. We will follow the thematic analysis approach developed by Thomas & Harden in 2008 to synthesise the qualitative data [38]. The analytic approach has three stages namely; (i) developing coding schemes, (ii) developing descriptive themes from the coding schemes, and (iii) generating analytic themes from the descriptive themes [38]. However, depending on the extracted data, we may follow other approaches such as meta-ethnography [39, 40], or "best fit" framework synthesis [41, 42] using the trajectories of women's abortion-

For peer review only - http://bmjopen.bmj.com/site/about/guidelines.xhtml

related care conceptual framework developed by Coast *et al.* [16] as a template. We will add other domains and sub-domains or modify existing ones, depending on the data we extract.

For the quantitative synthesis, we will extract the quantitative data into an excel sheet and then export these to the statistical software package Stata. For categorical variables, we will analyse pooled estimates using a random effects model. For continuous variables, we will calculate a pooled difference of means with 95% confidence intervals using a DerSimonian Laird random effects model. If the mean and standard deviation (SD) are not reported or are unavailable from the study authors, we will estimate them from sample size, median, range and/or interquartile range using the methods described by Wan *et al.* [43]. If we identify sufficient studies, we will conduct subgroup analysis by countries' abortion laws, World Bank economic group, and geographical regions. We will also conduct a sensitivity analysis excluding studies with low quality. We will assess heterogeneity using the I^2 test and publication bias using forest plots. We will only assess for publication bias if there are at least 10 studies included in the meta-analysis [44].

Following recommendations from the Cochrane Qualitative and Implementation Methods Group [45], we will report external validity of key qualitative synthesis using the Grades of Recommendation, Assessment, and Evaluation – Confidence in Evidence from Reviews of Qualitative Research (GRADE-CERQual) [46]. We will use the GRADE guidelines [46] to assess the quality of any quantitative findings.

Patient and public involvement

The data from the systematic review includes previously published data and will therefore not involve any patients or the public.

For peer review only - http://bmjopen.bmj.com/site/about/guidelines.xhtml

DISCUSSION

Today, systematic reviews have explored many aspects and issues related to abortion such as stigma [47], women's experiences of abortion [48, 49], contraception and abortion knowledge, attitudes and practices among adolescents from LMICs [50], methods of abortion [51], or prevalence of and risk factors for unsafe abortion (ongoing) [52]. Our systematic review will be one of the first to synthesise evidence relating to abortion decision-making processes in LMICs including abortion decision trajectories and factors influencing the choices of abortion trajectories. By focussing on LMICs, where nearly all unsafe abortions occur, we will use the evidence from the systematic review to develop an abortion decision-making model in LMICs to guide policy development and strategies to improve access to safe and faster abortion services and post abortion care by addressing barriers to safe abortion in LMICs.

Many abortion decision-making theories and models such as the conflict theory model of decision-making in abortion [53], the feminist theory [54, 55], or the autonomy and public health models [56] have shed light on the complexity of abortion decision-making processes. However, these models have tended to focus on specific aspects of the abortion decision-making process such as influence of circumstances preceding the pregnancy or locus of control on the decision to terminate pregnancy [53–56] yet abortion is such a complex, dynamic and iterative process influenced by various legal, socioeconomic, political and health system factors [9, 11, 12, 15–19]. Coast E. *et al.* developed a conceptual framework for understanding women's trajectories to abortion care that encompasses time-dependent abortion-specific experiences, and context-dependent individual and region-based experiences [16]. While its development involved consultative meetings with experts and review of studies published in English between 2011 and 2017, it was neither a comprehensive nor a systematic review of available knowledge and evidence [16]. Our systematic review of a wide range of bibliographic databases and grey

For peer review only - http://bmjopen.bmj.com/site/about/guidelines.xhtml

literature with no language restrictions including all articles published from 2000 will provide an in-depth understanding of the evidence relating to the complex decision-making processes and roles of various determinants that influence abortion trajectories of women in LMICs, which bear 97% of the burden of unsafe abortion and its complications [3].

Our review may have some limitations. Due to the sensitivity and scarcity of studies on abortion in some LMICs, few or no studies may be available from certain countries or regions where abortion is highly restricted. This may affect the generalisability of our results and data synthesis plan.

Ethics and dissemination

We did not require ethics approval for this systematic review. We will publish our findings in an open access peer-reviewed journal with a global health and maternal health readership. We will also present our findings at national and international scientific conferences.

Acknowledgements

We would like to thank Carolyn Smith, Library Manager/Outreach Librarian, Bodleian Health Care Libraries/Bodleian Libraries, Education Centre, Horton Hospital, University of Oxford for her help in developing the search strategy and training PL to conduct the searches.

Authors' contributions

PL, MN, JK, and CN conceived the idea, planned and designed the study protocol. PL wrote the first draft; SF, IC, JM, MN, JK, and CN all edited the draft and provided critical insights. All authors have approved and contributed to the final submitted manuscript.

Funding statement

For peer review only - http://bmjopen.bmj.com/site/about/guidelines.xhtml

This review is being supported by the Nuffield Department of Population Health (NDPH) at the University of Oxford which funds PL's doctoral research (Ref: MSD2021_119305) and the Medical Research Council (GCRF) Career Development Award (Grant Ref: MR/P022030/1) which funds MN. The funders had no role in the study protocol design, review process or writing of the paper. PL had full access to all the information for the paper and had the final responsibility for the decision to submit for publication.

Competing interests' statement

The authors declare no conflict of interest.

REFERENCES

[1] Bearak J, Popinchalk A, Ganatra B, et al. Unintended pregnancy and abortion by income, region, and the legal status of abortion: estimates from a comprehensive model for 1990–2019. *Lancet Glob Heal* 2020; 8: e1152–e1161.

[2] Tsuyoshi onda SSLRGS. LK. Uneven Progress and Unequal Access. *Abort Worldw 2017 Uneven Prog Unequal Access is available online at, Visit https//www.guttmacher.org/report/abortion-worldwide-2017* 2017; 1–68.

[3] Ganatra B, Gerdts C, Rossier C, et al. Articles Global, regional, and subregional classification of abortions by safety, 2010-14: estimates from a Bayesian hierarchical model. *www.thelancet.com*; 390. Epub ahead of print 2017. DOI: 10.1016/S0140-6736(17)31794-4.

[4] Say L, Chou D, Gemmill A, et al. Articles Global causes of maternal death: a WHO systematic analysis. 2014; 323.

[5] Vlassoff M, Shearer J, Walker D, et al. *Economic Impact of Unsafe Abortion-Related Morbidity and Mortality: Evidence and Estimation Challenges*. 2008.

[6] James SL, Abate D, Abate KH, et al. Global, regional, and national incidence, prevalence, and years lived with disability for 354 Diseases and Injuries for 195 countries and territories, 1990-2017: A systematic analysis for the Global Burden of Disease Study 2017. *Lancet* 2018; 392: 1789–1858.

[7] Yokoe R, Rowe R, Choudhury SS, et al. Unsafe abortion and abortion-related death among 1.8 million women in India. *BMJ Glob Heal* 2019; 4: 1–13.

[8] Prada E, Atuyambe LM, Blades NM, et al. Incidence of Induced Abortion in Uganda, 2013: New Estimates Since 2003. Epub ahead of print 2016. DOI:

For peer review only - http://bmjopen.bmj.com/site/about/guidelines.xhtml

10.1371/journal.pone.0165812.

[9] Jayaweera RT, Ngui FM, Hall KS, et al. Women's experiences with unplanned pregnancy and abortion in Kenya: A qualitative study. *PLoS One* 2018; 13: e0191412.

[10] Sundari Ravindran TK, Balasubramanian P. 'Yes' to abortion but 'no' to sexual rights: The paradoxical reality of married women in rural Tamil Nadu, India. *Reprod Health Matters* 2004; 12: 88–99.

[11] Freeman E, Coast E, Murray SF. Men's Roles in Women's Abortion Trajectories in Urban Zambia. *Int Perspect Sex Reprod Health* 2017; 43: 89–98.

[12] Engelbert Bain L, Zweekhorst MBM, Amoakoh-Coleman M, et al. To keep or not to keep? Decision making in adolescent pregnancies in Jamestown, Ghana. *PLoS One* 2019; 14: e0221789.

[13] Hajri S, Raifman S, Gerdts C, et al. 'This Is Real Misery': Experiences of Women Denied Legal Abortion in Tunisia. *PLoS One* 2015; 10: 1–16.

[14] Braam T, Hessini L. The power dynamics perpetuating unsafe abortion in Africa: a feminist perspective. *Afr J Reprod Health* 2004; 8: 43–51.

[15] Coast E, Murray SF. 'These things are dangerous': Understanding induced abortion trajectories in urban Zambia. *Soc Sci Med* 2016; 153: 201–209.

[16] Coast E, Norris AH, Moore AM, et al. Trajectories of women's abortion-related care: A conceptual framework. *Soc Sci Med* 2018; 200: 199–210.

[17] Engelbert Bain L, Amoakoh-Coleman M, Tiendrebeogo KST, et al. Attitudes towards abortion and decision-making capacity of pregnant adolescents: perspectives of medicine, midwifery and law students in Accra, Ghana. *Eur J Contracept Reprod Heal Care* 2020; 25: 151–158.

[18] Ouedraogo R, Senderowicz L, Ngbichi C. "I wasn't ready": abortion decision-making pathways in Ouagadougou, Burkina Faso. *Int J Public Health* 2020; 65: 477–486.

[19] Puri M, Ingham R, Matthews Z. Factors affecting abortion decisions among young couples in Nepal. *J Adolesc Heal Off Publ Soc Adolesc Med* 2007; 40: 535–542.

[20] Rehnström Loi U, Lindgren M, Faxelid E, et al. Decision-making preceding induced abortion: A qualitative study of women's experiences in Kisumu, Kenya 11 Medical and Health Sciences 1117 Public Health and Health Services. *Reprod Health* 2018; 15: 1–12.

[21] WHO. *Medical Management of Abortion*. 2018. Epub ahead of print 2018. DOI: 10.1097/AOG.0b013e3181e8b073.

[22] Moher D, Shamseer L, Clarke M, et al. *Preferred reporting items for systematic review and meta-analysis protocols (PRISMA-P) 2015 statement*. 2015. Epub ahead of print 2015. DOI: 10.1186/2046-4053-4-1.

[23] United Nations. *The Millennium Development Goals Report*. 2015.

For peer review only - http://bmjopen.bmj.com/site/about/guidelines.xhtml

[24] United Nations. List of Millennium Development Goals , and Goal 8 Targets and Indicators Goals 1 to 7 Goal 8 : Develop a global partnership for development Targets Indicators *. 100.

[25] World Bank Group. World Bank Country and Lending Groups – World Bank Data Help Desk. *2020*, https://datahelpdesk.worldbank.org/knowledgebase/articles/906519-world-bank-country-and-lending-groups (accessed 7 January 2021).

[26] Ma LL, Wang YY, Yang ZH, et al. Methodological quality (risk of bias) assessment tools for primary and secondary medical studies: What are they and which is better? *Mil Med Res* 2020; 7: 1–11.

[27] Sterne JAC, Savović J, Page MJ, et al. RoB 2: A revised tool for assessing risk of bias in randomised trials. *BMJ* 2019; 366: 1–8.

[28] Sterne JA, Hernán MA, Reeves BC, et al. ROBINS-I: A tool for assessing risk of bias in non-randomised studies of interventions. *BMJ* 2016; 355: 4–10.

[29] Stang A. Critical evaluation of the Newcastle-Ottawa scale for the assessment of the quality of nonrandomized studies in meta-analyses. *Eur J Epidemiol* 2010; 25: 603–605.

[30] Wells G, Shea B, O'Connell D, et al. The Newcastle-Ottawa Scale (NOS) for assessing the quality if nonrandomized studies in meta-analyses. *(Available from URL http//www.ohri.ca/programs/clinical_epidemiology/oxford.asp)*. Epub ahead of print 2012. DOI: 10.2307/632432.

[31] Moola S, Munn Z, Tufanaru C, et al. Checklist for Analytical Cross Sectional Studies. *Joanna Briggs Institute Reviewer's Manual*.

[32] Moola S, Munn Z, Sears K, et al. Conducting systematic reviews of association (etiology): The Joanna Briggs Institute's approach. *Int J Evid Based Healthc* 2015; 13: 163–169.

[33] Critical Appraisal Skills Programme. CASP Qualitative Checklist. *Casp*.

[34] Daly J, Willis K, Small R, et al. A hierarchy of evidence for assessing qualitative health research. *J Clin Epidemiol* 2007; 60: 43–49.

[35] Barnett-Page E, Thomas J. Methods for the synthesis of qualitative research: A critical review. *BMC Med Res Methodol*; 9. Epub ahead of print 2009. DOI: 10.1186/1471-2288-9-59.

[36] Noyes J, Booth A, Moore G, et al. Synthesising quantitative and qualitative evidence to inform guidelines on complex interventions: clarifying the purposes, designs and outlining some methods. *BMJ Glob Heal* 2019; 4: e000893.

[37] Hong QN, Pluye P, Bujold M, et al. Convergent and sequential synthesis designs: implications for conducting and reporting systematic reviews of qualitative and quantitative evidence. *Syst Rev* 2017; 6: 61.

[38] Thomas J, Harden A. Methods for the thematic synthesis of qualitative research in systematic reviews. *BMC Med Res Methodol* 2008; 8: 1–10.

For peer review only - http://bmjopen.bmj.com/site/about/guidelines.xhtml

[39] Atkins S, Lewin S, Smith H, et al. Conducting a meta-ethnography of qualitative literature: Lessons learnt. *BMC Med Res Methodol* 2008; 8: 1–10.

[40] France EF, Uny I, Ring N, et al. A methodological systematic review of meta-ethnography conduct to articulate the complex analytical phases 11 Medical and Health Sciences 1117 Public Health and Health Services. *BMC Med Res Methodol* 2019; 19: 1–18.

[41] Carroll C, Booth A, Cooper K. A worked example of 'best fit' framework synthesis: A systematic review of views concerning the taking of some potential chemopreventive agents. *BMC Med Res Methodol*; 11. Epub ahead of print 2011. DOI: 10.1186/1471-2288-11-29.

[42] Carroll C, Booth A, Leaviss J, et al. 'best fit' framework synthesis: Refining the method. *BMC Med Res Methodol*; 13. Epub ahead of print 2013. DOI: 10.1186/1471-2288-13-37.

[43] Wan X, Wang W, Liu J, et al. Estimating the sample mean and standard deviation from the sample size, median, range and/or interquartile range. *BMC Med Res Methodol* 2014; 14: 1–13.

[44] Kimball ABBAB, Guérin A, Tsaneva M, et al. Cochrane Handbook for Systematic Reviews of Interventions Version 5.1.0 [updated March 2011]. The Cochrane Collaboration, 2011. Available from www.handbook.cochrane.org. *Br J Dermatol*.

[45] Noyes J, Booth A, Flemming K, et al. Cochrane Qualitative and Implementation Methods Group guidance series—paper 3: methods for assessing methodological limitations, data extraction and synthesis, and confidence in synthesized qualitative findings. *J Clin Epidemiol* 2018; 97: 49–58.

[46] Lewin S, Booth A, Glenton C, et al. Applying GRADE-CERQual to qualitative evidence synthesis findings: Introduction to the series. *Implement Sci* 2018; 13: 1–10.

[47] Hanschmidt F, Linde K, Hilbert A, et al. Abortion Stigma: A Systematic Review. *Perspect Sex Reprod Health* 2016; 48: 169–177.

[48] Lie MLS, Robson SC, May CR. Experiences of abortion: A narrative review of qualitative studies. *BMC Health Serv Res* 2008; 8: 1–9.

[49] Barr-Walker J, Jayaweera RT, Ramirez AM, et al. Experiences of women who travel for abortion: A mixed methods systematic review. *PLoS One* 2019; 14: 1–26.

[50] Munakampe MN, Zulu JM, Michelo C. Contraception and abortion knowledge, attitudes and practices among adolescents from low and middle-income countries: a systematic review. *BMC Health Serv Res* 2018; 18: 909.

[51] Wen J, Cai QY, Deng F, et al. Manual versus electric vacuum aspiration for first-trimester abortion: A systematic review. *BJOG An Int J Obstet Gynaecol* 2008; 115: 5–13.

[52] Gebremedhin M, Semahegn A, Usmael T, et al. Unsafe abortion and associated factors among reproductive aged women in Sub-Saharan Africa: a protocol for a systematic review and meta-analysis. *Syst Rev* 2018; 7: 130.

For peer review only - http://bmjopen.bmj.com/site/about/guidelines.xhtml

[53] Friedlander ML, Kaul TJ, Stimel CA. Abortion: Predicting the complexity of the decision-making process. *Women Heal* 1984; 9: 43–54.

[54] Marecek J, Macleod C, Hoggart L. Abortion embedded and embodied in social relations: Challenges for feminist psychology. *Fem Psychol* 2017; 27: 133–143.

[55] Marecek J, Macleod C, Hoggart L. Abortion in legal, social, and healthcare contexts. *Fem Psychol* 2017; 27: 4–14.

[56] Manian M. The Irrational Woman: Informed Consent and Abortion Decision-Making. *Duke J Gend Law Policy*.

Word count: 3013

For peer review only

For peer review only - http://bmjopen.bmj.com/site/about/guidelines.xhtml

[i]PRISMA-P (Preferred Reporting Items for Systematic review and Meta-Analysis Protocols) 2015 checklist: recommended items to address in a systematic review protocol*

Section and topic	Item No	Checklist item	Self-Evaluation
**ADMINISTRATIVE INFORMATION**			
Title:			
Identification	1a	Identify the report as a protocol of a systematic review	**YES, identified**
Update	1b	If the protocol is for an update of a previous systematic review, identify as such	**Not applicable**
Registration	2	If registered, provide the name of the registry (such as PROSPERO) and registration number	**CRD42021224719**
Authors:			
Contact	3a	Provide name, institutional affiliation, e-mail address of all protocol authors; provide physical mailing address of corresponding author	**YES, it is provided**
Contributions	3b	Describe contributions of protocol authors and identify the guarantor of the review	**YES, this is provided**
Amendments	4	If the protocol represents an amendment of a previously completed or published protocol, identify as such and list changes; otherwise, state plan for documenting important protocol amendments	**Not applicable**
Support:			
Sources	5a	Indicate sources of financial or other support for the review	**Yes**
Sponsor	5b	Provide name for the review funder and/or sponsor	**Yes**
Role of sponsor or funder	5c	Describe roles of funder(s), sponsor(s), and/or institution(s), if any, in developing the protocol	**Yes**
**INTRODUCTION**			
Rationale	6	Describe the rationale for the review in the context of what is already known	**Yes**
Objectives	7	Provide an explicit statement of the question(s) the review will address with reference to participants, interventions, comparators, and outcomes (PICO)	**Yes**
**METHODS**			
Eligibility criteria	8	Specify the study characteristics (such as PICO, study design, setting, time frame) and report characteristics (such as years considered, language, publication status) to be used as criteria for eligibility for the review	**Yes**
Information sources	9	Describe all intended information sources (such as electronic databases, contact with study authors, trial registers or other grey literature sources) with planned dates of coverage	**Yes**
Search strategy	10	Present draft of search strategy to be used for at least one electronic database, including planned limits, such that it could be repeated	**Yes (supplementary file 2)**

For peer review only

Study records:			
Data management	11a	Describe the mechanism(s) that will be used to manage records and data throughout the review	**Yes**
Selection process	11b	State the process that will be used for selecting studies (such as two independent reviewers) through each phase of the review (that is, screening, eligibility and inclusion in meta-analysis)	**Yes**
Data collection process	11c	Describe planned method of extracting data from reports (such as piloting forms, done independently, in duplicate), any processes for obtaining and confirming data from investigators	**Yes**
Data items	12	List and define all variables for which data will be sought (such as PICO items, funding sources), any pre-planned data assumptions and simplifications	**Yes**
Outcomes and prioritization	13	List and define all outcomes for which data will be sought, including prioritization of main and additional outcomes, with rationale	**Yes**
Risk of bias in individual studies	14	Describe anticipated methods for assessing risk of bias of individual studies, including whether this will be done at the outcome or study level, or both; state how this information will be used in data synthesis	**Yes**
Data synthesis	15a	Describe criteria under which study data will be quantitatively synthesised	**Yes**
	15b	If data are appropriate for quantitative synthesis, describe planned summary measures, methods of handling data and methods of combining data from studies, including any planned exploration of consistency (such as I^2, Kendall's τ)	**Yes**
	15c	Describe any proposed additional analyses (such as sensitivity or subgroup analyses, meta-regression)	**Yes**
	15d	If quantitative synthesis is not appropriate, describe the type of summary planned	**Yes**
Meta-bias(es)	16	Specify any planned assessment of meta-bias(es) (such as publication bias across studies, selective reporting within studies)	**Yes**
Confidence in cumulative evidence	17	Describe how the strength of the body of evidence will be assessed (such as GRADE)	**Yes**

*** It is strongly recommended that this checklist be read in conjunction with the PRISMA-P Explanation and Elaboration (cite when available) for important clarification on the items. Amendments to a review protocol should be tracked and dated. The copyright for PRISMA-P (including checklist) is held by the PRISMA-P Group and is distributed under a Creative Commons Attribution Licence 4.0.**

From: Shamseer L, Moher D, Clarke M, Ghersi D, Liberati A, Petticrew M, Shekelle P, Stewart L, PRISMA-P Group. Preferred reporting items for systematic review and meta-analysis protocols (PRISMA-P) 2015: elaboration and explanation. BMJ. 2015 Jan 2;349(jan02 1):g7647.

---
i

For peer review only

Abortion decision-making trajectories and determinants in low- and middle-income countries: a protocol for mixed methods systematic review and meta-analysis
Preliminary sample search for Ovid Medline: Search date 28/12/2020

#	Search	Results
1	(afghanistan or albania or algeria or american samoa or angola or "antigua and barbuda" or antigua or barbuda or argentina or armenia or armenian or aruba or azerbaijan or bahrain or bangladesh or barbados or republic of belarus or belarus or byelarus or belorussia or byelorussian or belize or british honduras or benin or dahomey or bhutan or bolivia or "bosnia and herzegovina" or bosnia or herzegovina or botswana or bechuanaland or brazil or brasil or bulgaria or burkina faso or burkina fasso or upper volta or burundi or urundi or cabo verde or cape verde or cambodia or kampuchea or khmer republic or cameroon or cameron or cameroun or central african republic or ubangi shari or chad or chile or china or colombia or comoros or comoro islands or iles comores or mayotte or democratic republic of the congo or democratic republic congo or congo or zaire or costa rica or "cote d'ivoire" or "cote d' ivoire" or cote divoire or cote d ivoire or ivory coast or croatia or cuba or cyprus or czech republic or czechoslovakia or djibouti or french somaliland or dominica or dominican republic or ecuador or egypt or united arab republic or el salvador or equatorial guinea or spanish guinea or eritrea or estonia or eswatini or swaziland or ethiopia or fiji or gabon or gabonese republic or gambia or "georgia (republic)" or georgian or ghana or gold coast or gibraltar or greece or grenada or guam or guatemala or guinea or guinea bissau or guyana or british guiana or haiti or hispaniola or honduras or hungary or india or indonesia or timor or iran or iraq or isle of man or jamaica or jordan or kazakhstan or kazakh or kenya or "democratic people's republic of korea" or republic of korea or north korea or south korea or korea or kosovo or kyrgyzstan or kirghizia or kirgizstan or kyrgyz republic or kirghiz or laos or lao pdr or "lao people's democratic republic" or latvia or lebanon or lebanese republic or lesotho or basutoland or liberia or libya or libyan arab jamahiriya or lithuania or macau or macao or republic of north macedonia or macedonia or madagascar or malagasy republic or malawi or nyasaland or malaysia or malay federation or malaya federation or maldives or indian ocean islands or indian ocean or mali or malta or micronesia or federated states of micronesia or kiribati or marshall islands or nauru or northern mariana islands or palau or tuvalu or mauritania or mauritius or mexico or moldova or moldovian or mongolia or montenegro or morocco or ifni or mozambique or portuguese east africa or myanmar or burma or namibia or nepal or netherlands antilles or nicaragua or niger or nigeria or oman or muscat or pakistan or panama or papua new guinea or new guinea or paraguay or peru or philippines or philipines or phillipines or phillippines or poland or "polish people's republic" or portugal or portuguese republic or puerto rico or romania or russia or russian federation or ussr or soviet union or union of soviet socialist republics or rwanda or ruanda or samoa or pacific islands or polynesia or samoan islands or navigator island or navigator islands or "sao tome and principe" or saudi arabia or senegal or serbia or seychelles or sierra leone or slovakia or slovak republic or slovenia or melanesia or solomon island or solomon islands or norfolk island or norfolk islands or somalia or south africa or south sudan or sri lanka or ceylon or "saint kitts and nevis" or "st. kitts and nevis" or saint lucia or "st. lucia" or "saint vincent and the grenadines" or	1981780

For peer review only

	saint vincent or "st. vincent" or grenadines or sudan or suriname or surinam or dutch guiana or netherlands guiana or syria or syrian arab republic or tajikistan or tadjikistan or tadzhikistan or tadzhik or tanzania or tanganyika or thailand or siam or timor leste or east timor or togo or togolese republic or tonga or "trinidad and tobago" or trinidad or tobago or tunisia or turkey or turkmenistan or turkmen or uganda or ukraine or uruguay or uzbekistan or uzbek or vanuatu or new hebrides or venezuela or vietnam or viet nam or middle east or west bank or gaza or palestine or yemen or yugoslavia or zambia or zimbabwe or northern rhodesia or global south or africa south of the sahara or sub-saharan africa or subsaharan africa or africa, central or central africa or africa, northern or north africa or northern africa or magreb or maghrib or sahara or africa, southern or southern africa or africa, eastern or east africa or eastern africa or africa, western or west africa or western africa or west indies or indian ocean islands or caribbean or central america or latin america or "south and central america" or south america or asia, central or central asia or asia, northern or north asia or northern asia or asia, southeastern or southeastern asia or south eastern asia or southeast asia or south east asia or asia, western or western asia or europe, eastern or east europe or eastern europe or developing country or developing countries or developing nation? or developing population? or developing world or less developed countr* or less developed nation? or less developed population? or less developed world or lesser developed countr* or lesser developed nation? or lesser developed population? or lesser developed world or under developed countr* or under developed nation? or under developed population? or under developed world or underdeveloped countr* or underdeveloped nation? or underdeveloped population? or underdeveloped world or middle income countr* or middle income nation? or middle income population? or low income countr* or low income nation? or low income population? or lower income countr* or lower income nation? or lower income population? or underserved countr* or underserved nation? or underserved population? or underserved world or under served countr* or under served nation? or under served population? or under served world or deprived countr* or deprived nation? or deprived population? or deprived world or poor countr* or poor nation? or poor population? or poor world or poorer countr* or poorer nation? or poorer population? or poorer world or developing econom* or less developed econom* or lesser developed econom* or under developed econom* or underdeveloped econom* or middle income econom* or low income econom* or lower income econom* or low gdp or low gnp or low gross domestic or low gross national or lower gdp or lower gnp or lower gross domestic or lower gross national or lmic or lmics or third world or lami countr* or transitional countr* or emerging economies or emerging nation?).ti,ab,sh,kw.	
2	exp Abortion, Induced/	40425
3	abortion.mp.	88886
4	pregnancy termination.mp.	2413
5	2 or 3 or 4	90396
6	decision making.mp.	222374
7	exp Decision Making/	206294
8	6 or 7	327808

For peer review only

9	1 and 5 and 8	496
10	limit 9 to (humans and yr="2000 -Current")	321

For peer review only

BMJ Open

Abortion decision-making trajectories and factors influencing such trajectories in low- and middle-income countries: a protocol for mixed methods systematic review

Journal:	*BMJ Open*
Manuscript ID	bmjopen-2021-049507.R1
Article Type:	Protocol
Date Submitted by the Author:	09-Sep-2021
Complete List of Authors:	Lokubal, Paul; University of Oxford, Nuffield Department of Population Health (NDPH)<br>Frischer, Sandrena Ruth; Partners in Health, Monrovia<br>Corcuera, Ines; Chelsea and Westminster Hospital NHS Foundation Trust<br>Balil, Jessica ; MSI Reproductive Choices UK<br>Nalwadda Kayemba, Christine ; Karolinska Institute/Makerere University<br>Kurinczuk, Jennifer; University of Oxford, National Perinatal Epidemiology Unit<br>Nair, Manisha; University of Oxford, NPEU, Nuffield Department of Population Health
**Primary Subject Heading**:	Global health
Secondary Subject Heading:	Sexual health
Keywords:	PUBLIC HEALTH, Health policy < HEALTH SERVICES ADMINISTRATION & MANAGEMENT, Maternal medicine < OBSTETRICS

SCHOLARONE™
Manuscripts

I, the Submitting Author has the right to grant and does grant on behalf of all authors of the Work (as defined in the below author licence), an exclusive licence and/or a non-exclusive licence for contributions from authors who are: i) UK Crown employees; ii) where BMJ has agreed a CC-BY licence shall apply, and/or iii) in accordance with the terms applicable for US Federal Government officers or employees acting as part of their official duties; on a worldwide, perpetual, irrevocable, royalty-free basis to BMJ Publishing Group Ltd ("BMJ") its licensees and where the relevant Journal is co-owned by BMJ to the co-owners of the Journal, to publish the Work in this journal and any other BMJ products and to exploit all rights, as set out in our licence.

The Submitting Author accepts and understands that any supply made under these terms is made by BMJ to the Submitting Author unless you are acting as an employee on behalf of your employer or a postgraduate student of an affiliated institution which is paying any applicable article publishing charge ("APC") for Open Access articles. Where the Submitting Author wishes to make the Work available on an Open Access basis (and intends to pay the relevant APC), the terms of reuse of such Open Access shall be governed by a Creative Commons licence – details of these licences and which Creative Commons licence will apply to this Work are set out in our licence referred to above.

Other than as permitted in any relevant BMJ Author's Self Archiving Policies, I confirm this Work has not been accepted for publication elsewhere, is not being considered for publication elsewhere and does not duplicate material already published. I confirm all authors consent to publication of this Work and authorise the granting of this licence.

For peer review only

Abortion decision-making trajectories and factors influencing such trajectories in low- and middle-income countries: a protocol for mixed methods systematic review.

Co-authors:

1. Paul Lokubal, MSc; National Perinatal Epidemiology Unit (NPEU), Nuffield Department of Population Health (NDPH), University of Oxford, Old Road Campus, Headington, Oxford OX3 7LF, Oxford, UK
2. Sandrena Ruth Frischer, MSc; Partners in Health, Monrovia, Liberia
3. Ines Corcuera, MSc; Chelsea and Westminster Hospital, NHS Foundation Trust, 369 Fulham Road, Chelsea, London SW10 9NH, London, UK
4. Jessica Macias Balil, MSc; MSI Reproductive Choices, Conway St, Fitzroy Square, Fitzrovia, London W1T 6LP, London, UK
5. Christine Nalwadda Kayemba, PhD; Department of Community Health and Behavioural Sciences, School of Public, College of Health Sciences, Makerere University, Kampala, Uganda
6. Jennifer J Kurinczuk, MD; National Perinatal Epidemiology Unit (NPEU), Nuffield Department of Population Health (NDPH), University of Oxford, Old Road Campus, Headington, Oxford OX3 7LF, Oxford, UK
7. Manisha Nair, DPhil; National Perinatal Epidemiology Unit (NPEU), Nuffield Department of Population Health (NDPH), University of Oxford, Old Road Campus, Headington, Oxford OX3 7LF, Oxford, UK

Corresponding author: Paul Lokubal, DPhil Student; National Perinatal Epidemiology Unit (NPEU), Nuffield Department of Population Health (NDPH), University of Oxford, Old Road Campus, Headington, Oxford OX3 7LF, Oxford, UK. paul.lokubal@ndph.ox.ac.uk

Systematic Registration: PROSPERO 2021: CRD42021224719

Key words: abortion, decision-making, abortion trajectories, low- and middle-income countries, mixed methods, systematic review, meta-analysis.

For peer review only - http://bmjopen.bmj.com/site/about/guidelines.xhtml

ABSTRACT

Introduction: Globally, about half of all pregnancies are unintended and/or unwanted and three-fifths of these end in induced abortion. When faced with a choice to terminate pregnancy, women's abortion decision-making processes are often complex and multiphasic and maybe amplified in low- and middle-income countries (LMICs) which bear the major burden of abortion-related morbidity and mortality. Our review aims to 1) describe abortion decision-making trajectories for women in LMICs and 2) investigate factors influencing the choice of abortion decision-making trajectories in LMICs.

Methods and Analysis: We will search and retrieve published and unpublished qualitative, quantitative and mixed methods, community and/or hospital-based studies conducted in LMICs from January 1st, 2000 up to February 16th, 2021. We will search Ovid Medline, Ovid EMBASE, Ovid PsychInfo, Ovid Global Health, Web of Science (including Social Science Citation Index), Scopus, IBSS, CINAHL via EBSCO, WHO Global Index Medicus, the Cochrane Library, WHO website, ProQuest, and Google Scholar. We will search reference lists of eligible studies and contact experts for additional data/ information, if required. We will extract all relevant data to answer our research questions and assess study quality using the appropriate appraisal tools. Depending on the extracted data, our analysis will use sequential or convergent synthesis methods proposed by Hong *et al*. For qualitative studies, we will synthesise evidence using thematic synthesis, meta-ethnography or "best-fit" framework synthesis and for quantitative findings, we will do descriptive synthesis and/or meta-analysis. We will do sensitivity analyses and assess confidence in our findings using GRADE-CERQUal for qualitative findings and GRADE for quantitative findings.

Ethics and Dissemination: We did not require ethics approval for this systematic review. We will publish our findings in an open access peer-reviewed journal with global and maternal health readership. We will also present our findings at national and international scientific conferences.

For peer review only - http://bmjopen.bmj.com/site/about/guidelines.xhtml

Strengths and limitations of this study

- The review is one of the first to synthesize evidence on abortion decision-making processes in LMICs including abortion decision trajectories and factors influencing their choices.
- The review includes multiple databases, grey literature with no language restrictions and covers articles published from 2000 onwards up to February 16th 2021 in order to capture the contemporary abortion decision-making process.
- The systematic review will be conducted following the PRISMA guidelines, this includes the use of at least two reviewers to independently search, screen and select, extract data, and assess quality of included studies.
- Due to the sensitivity and scarcity of studies on abortion in some LMICs, few or no studies may be available from certain countries or regions where abortion is highly restricted which may affect our results and data synthesis plan.

INTRODUCTION

Globally, an estimated 48% (121 million) of all pregnancies each year from 2015-2019 were unintended and/or unwanted and 61% (73 million) of these ended in induced abortion [1, 2]. The proportion of unintended and/or unwanted pregnancies that end in induced abortion is similar between low-income countries (LICs) and high- income countries (HICs) (40% and 43% respectively) but higher in middle-income countries (MICs) (66)% [1]. Between 2010 and 2014, 45% of all abortions were estimated to be unsafe with 97% occurring in low- and middle- income countries (LMICs) [3]. The proportion of all abortions that are unsafe is about four times higher (49.5%) in LMICs compared to HICs (12.5%) [3]. The proportion of unsafe abortions is 0.9% in North America, 2.1% in Northern Europe, 37.8% in Asia, 75.6% in Africa, and 76.4% in Latin America [3]. Unsafe abortion and its complications are a major cause of avoidable maternal

For peer review only - http://bmjopen.bmj.com/site/about/guidelines.xhtml

deaths and morbidity globally, accounting for 4.7-13.2% of all maternal deaths [4], USD 553 million in treatment costs in LMICs [5], and 18,100 years lived with disability (YLDs) [6]. Despite accounting for only 29% of all unsafe abortions globally, 62% of all abortion-related deaths occur in Africa [3].

While the differences in unsafe abortion rates and related morbidity and mortality differ markedly according to a country's Gross Domestic Product (GDP), the overall induced abortion rates are somewhat similar worldwide [1, 2]. Globally, the highest overall abortion rates are seen in MICs and the lowest in HICs; the rates per 1,000 women aged 15-49 are 44 in MICs, 38 in LICs and 15 in HICs [1–3]. Generally, while restrictive abortion laws make most abortions unsafe [3], the overall abortion rates are similar in countries with varying abortion laws [1, 2]. However, in LMICs unsafe abortion rates are similar regardless of a country's abortion laws [7, 8]. The majority of induced abortions are for unwanted pregnancies due to failure or non-use of contraception, rape, defilement, or incest [2]. However, even planned pregnancies can become unwanted due to changes in circumstances during pregnancy including health concerns if the pregnancy is continued to term [2]. Other reasons for abortion include: financial concerns, parenting readiness, need to space or limit childbirths, influence from significant others (such as partners and family), lack of support for the pregnancy from partners or family members, career and education goals, and stigmatised pregnancies such as teenage or out of wedlock pregnancies [9–13].

Due to the sensitivity and the socioeconomic and power dynamics involved in abortion [14], abortion decision-making trajectories are often complex, iterative, multiphasic, dynamic, context-specific and may involve periods of intense negotiations between the woman and the significant others [9–12, 15–19]. According to Coast *et al.*, abortion decision-making trajectories are "*the processes and transitions occurring over time for a pregnancy that ends in abortion"* [16]. The circumstances surrounding a woman's decision to seek an abortion can be time-specific and

For peer review only - http://bmjopen.bmj.com/site/about/guidelines.xhtml

variable [18]. Women may “suffer in silence” due to uncertainty on who to talk to about the decision to terminate a pregnancy and their reactions to such a decision [20]. The abortion trajectories chosen may affect the safety of the abortion and access to post-abortion care [15, 20]. The particular trajectory taken is influenced by various legal, socioeconomic, demographic, and cultural factors such as financial stability, relationship stability, influence of significant others, risk perceptions, stigma, knowledge of abortion laws, and availability and access to abortion services [9, 11, 12, 15–19]. Additionally, the increasing availability and use of misoprostol to terminate pregnancy means that women can now access abortion services outside formal health care systems [21] thus bypassing legal restrictions in settings where abortion is illegal [22]

Rationale for the systematic review

With 97% of all unsafe abortions occurring in LMICs [3], it is important to synthesise evidence on the abortion decision-making processes in these settings. The aim is to conduct a systematic review to synthesize the evidence relating to abortion decision-making trajectories and their determinants in LMICs. The review will help to visualise the complex decision-making trajectories which in turn could bring to light the unrecognised factors that contribute to unsafe abortion and pave way for further research and policy actions to address unsafe abortion in LMIC settings.

Review questions

The questions address by this systematic review are:

1. What are the abortion decision-making trajectories for women seeking abortion in LMICs?
2. For women in LMICs, what factors influence the choice of these abortion trajectories?

For peer review only - http://bmjopen.bmj.com/site/about/guidelines.xhtml

METHODOLOGY

Development of review protocol and registration

We followed the guidelines set out in the preferred reporting items for systematic review and meta-analysis protocol (PRISMA-P) 2015 statement [23] to develop the protocol. We completed the PRISMA-P checklist (supplementary file 1). The review protocol has been registered with international prospective register of systematic reviews (PROSPERO) with systematic review registration number CRD42021224719.

Searches

The search strategy will be developed with the assistance of an information librarian. The first author (PL) will search the following electronic bibliographic databases: Ovid Medline, Ovid EMBASE, Ovid PsychInfo, Ovid Global Health, Web of Science (including Social Science Citation Index), Scopus, IBSS, CINAHL via EBSCO, WHO Global Index Medicus, and the Cochrane Library. PL will also search grey literature sources including ProQuest, Google Scholar and the WHO website.

All references of all included articles will be checked for additional articles that may have been missed from earlier searches. In addition, we will also contact experts – with experience in the field of abortion in LMICs – for any additional articles. We will limit our search strategy to articles published from January 1st, 2000. Due to time constraints, the last search date for articles will be on February 16th, 2021. The year 2000 has been chosen because it marked the start of the Millennium Development Goals (MDGs) which included a global commitment to reduce by 75%, between 1990 and 2015, the maternal mortality ratio [24, 25]. Since then, many countries have liberalised abortion laws or decriminalised abortion [2].

For peer review only - http://bmjopen.bmj.com/site/about/guidelines.xhtml

There will be no language restrictions in order to maximise the relevant articles from LMICs. The search strings will be composed of the following three key concepts and their synonyms: “abortion,” “decision-making”, “developing countries” and will be written with Boolean terms. We will modify the search strings depending on database requirements and use both key words in English and medical subject headings (MeSH) in the search process. We will use the search filters for LMICs from Cochrane (https://epoc.cochrane.org/lmic-filters). We will create email alerts for any new relevant articles published and re-run the searches before the final analysis to identify and retrieve any further eligible studies for inclusion. We will maintain records of all searches for each database. A sample of the search strategy from Ovid Medline that was generated by the Librarian and PL is attached (supplementary file 2).

Eligibility Criteria

Inclusion and exclusion criteria for studies

All eligible observational studies (cross-sectional, case-control, and cohort), surveys, technical reports, and intervention studies will be included in the systematic review. Although we will exclude trial registrations, systematic review protocols, systematic reviews, case series, conference abstracts, case reports, policy analyses, commentaries, conceptual frameworks, and editorials from the review, we will cross-check their reference lists to identify and retrieve, if any, further articles for inclusion. We will consider all relevant published and unpublished (grey literature) quantitative, qualitative, and mixed methods studies restricted to humans.

Participants/Population

For the studies to be included, the population studied must be women who had an induced abortion and/or other actors such as abortion care providers whether skilled or unskilled, formal or informal and women’s male partners who were directly involved in the abortion decision-

For peer review only - http://bmjopen.bmj.com/site/about/guidelines.xhtml

making process for that induced abortion. We shall exclude studies that focus only on women with spontaneous abortions or miscarriages, or reports or opinions of health care providers, and policy makers on abortion.

Intervention(s), exposure(s)

There is no intervention for our review but our focus is to understand abortion decision-making processes in LMICs in women who undergo induced abortions. We will focus on abortion decision trajectories and factors influencing the choice of such trajectories.

Comparators

While having a comparator is not essential for this review, studies such as observational studies having comparison groups will not be excluded on the basis of having control or comparator groups.

Outcomes

The main outcomes of this review include abortion trajectories and factors influencing choices of abortion trajectories in LMICs.

Context or study settings

We will consider only studies conducted in LMICs as defined by World Bank [26] irrespective of the legal status of and policy environment on abortion. We will include all relevant community and/or facility-based studies that used either primary or secondary data. We will exclude animal studies.

Study screening and selection

For peer review only - http://bmjopen.bmj.com/site/about/guidelines.xhtml

We will use Covidence software to screen and select eligible studies. The study screening and selection will take place in two stages with PL involved in screening all articles from the search strategy while second author (SF) will screen 40% of all included articles and IC and JMB (third and fourth authors) will screen 30% each. In the first stage, the reviewers will independently screen all titles and abstracts based on inclusion criteria. All four reviewers will regularly discuss results to verify the selection process and include all relevant articles for full text-review. In the second stage, the two groups of reviewers will independently read the full texts of all selected articles and include only those mentioning either of the key outcomes including trajectories of abortion decision-making or determinants of such trajectories. For the full-text screening, the authors will resolve any disagreements by consensus or by consulting the senior author (MN) and/or the coinvestigator group. We will chart the results of the screening and selection process on the PRISMA flow diagram.

Data extraction

We will use the Covidence systematic review software to extract data and assess study quality. We will extract the following information: study aim(s); study setting (including location(s) and year(s)); inclusion/exclusion criteria and participant characteristics; study methodology (including study design, sample size, data collection and analytical methods); results (including frequencies, effect sizes, themes, quotes, author interpretations or explanations); strengths and limitations; reviewer comments; and all information needed to assess the risk of bias. The extraction will be done by PL (all articles), with SF, IC and JMB being second assessors. Two authors will extract the data independently and resolve discrepancies through discussion, involving another reviewer (MN) when necessary. We will contact authors for any missing, uncertain, or incomplete information and if there is no response within 2 weeks, we may exclude those articles based on missing information. We will first pilot our data extraction process,

For peer review only - http://bmjopen.bmj.com/site/about/guidelines.xhtml

independently and in duplicate, on five articles and make further refinements as needed. Depending upon the extracted data, we may generate single or separate data extraction templates for qualitative and quantitative findings.

Risk of bias (quality) assessment

Each article will be assessed by two reviewers, with PL reviewing all articles and SF, IC, and JMB being the second assessors. We anticipate that the majority of studies will be qualitative with few or no observational studies and experimental studies. We will use the most appropriate quality assessment tools for the studies included [27]. The assessment will therefore be based on the articles included and will involve at least two reviewers assessing each article independently.

We will use the revised 2019 version of the Cochrane risk of bias tool (RoB 2) [28] to assess randomised controlled trials (RCTs) if we find any. To assess the quality of non-randomised controlled trials (non-RCTs), we will use the Risk Of Bias In Non-randomised Studies – of Interventions (ROBINS-I) [29]. We will rate the overall quality assessment as low, moderate, serious, critical or no information provided [29].

For cohort and case-control studies, we will use the Newcastle-Ottawa Scale (NOS) [30, 31]. This tool is best for cohort and case control studies as it allows user modification [27]. For analytical or descriptive cross-sectional studies, we will use the Joanna Briggs Institute (JBI) assessment tool [32, 33].

For qualitative studies, we will use the critical appraisal skills programme (CASP) appraisal checklist for qualitative studies and assign each paper an overall quality ranking of “low,” “medium,” or “high” [34].

For peer review only - http://bmjopen.bmj.com/site/about/guidelines.xhtml

Each reviewer will independently assess and rate each included study using the relevant quality assessment tool. We will discuss the quality assessment and risk of bias assessment findings and resolve any disagreements by consensus or by involving the senior author (MN) if necessary. For “poor” quality qualitative studies, we will contact the authors for more information, a standard practice for assessing quality of qualitative studies [35]. We will not exclude any studies based on quality assessment [36]. We will present results of quality assessment in tabular form with comments or explanations.

Strategy for data synthesis

While no widely accepted approach is available for synthesising a mixed methods systematic review, and any approach chosen depends upon the type of studies (qualitative, quantitative or mixed methods) and the purpose of the research [37], we will analyse the data on the basis of the findings from our search. We anticipate that there will be mainly qualitative studies and the quantitative studies available may not be sufficient for meta-analysis or the findings are likely to be heterogeneous. If this is the case, we will provide a narrative summary of the quantitative findings. However, if there are sufficient quantitative studies, we will follow one of the two approaches in the synthesis as suggested by Hong *et al.* [38]: (1) sequential synthesis design involving two phases: in phase one, we will first identify the main themes or components of the research questions using qualitative synthesis. In phase two, we will analyse quantitative studies to quantify the effect of each component or theme; or (2) convergent synthesis design – we will analyse qualitative and quantitative studies separately and integrate the findings at the results or discussion stage. We will use the results to develop an abortion decision-making model for women in LMICs from our analysis.

For qualitative analysis, we will upload extracted information into NVivo software to support the qualitative analysis. We will follow the thematic analysis approach developed by Thomas &

For peer review only - http://bmjopen.bmj.com/site/about/guidelines.xhtml

Harden in 2008 to synthesise the qualitative data [39]. The analytic approach has three stages namely; (i) developing coding schemes, (ii) developing descriptive themes from the coding schemes, and (iii) generating analytic themes from the descriptive themes [39]. However, depending on the extracted data, we may follow other approaches such as meta-ethnography [40, 41], or "best fit" framework synthesis [42, 43] using the trajectories of women's abortion-related care conceptual framework developed by Coast *et al.* [16] as a template. We will add other domains and sub-domains or modify existing ones, depending on the data we extract.

For the quantitative synthesis, we will extract the quantitative data into an excel sheet and then export these to the statistical software package Stata. For categorical variables, we will analyse pooled estimates using a random effects model. For continuous variables, we will calculate a pooled difference of means with 95% confidence intervals using a DerSimonian Laird random effects model. If the mean and standard deviation (SD) are not reported or are unavailable from the study authors, we will estimate them from sample size, median, range and/or interquartile range using the methods described by Wan *et al.* [44]. If we identify sufficient studies, we will conduct subgroup analysis by countries' abortion laws, World Bank economic group, and geographical regions. We will also conduct a sensitivity analysis excluding studies with low quality. We will assess heterogeneity using the I^2 test and publication bias using forest plots. We will only assess for publication bias if there are at least 10 studies included in the meta-analysis [45].

Following recommendations from the Cochrane Qualitative and Implementation Methods Group [46], we will report external validity of key qualitative synthesis using the Grades of Recommendation, Assessment, and Evaluation – Confidence in Evidence from Reviews of Qualitative Research (GRADE-CERQUal) [47]. We will use the GRADE guidelines to assess the quality of any quantitative findings [48].

For peer review only - http://bmjopen.bmj.com/site/about/guidelines.xhtml

Patient and public involvement

The data from the systematic review includes previously published data and will therefore not involve any patients or the public.

Ethics and dissemination

We did not require ethics approval for this systematic review. We will publish our findings in an open access peer-reviewed journal with a global health and maternal health readership. We will also present our findings at national and international scientific conferences.

Acknowledgements

We would like to thank Carolyn Smith, Library Manager/Outreach Librarian, Bodleian Health Care Libraries/Bodleian Libraries, Education Centre, Horton Hospital, University of Oxford for her help in developing the search strategy and training PL to conduct the searches.

Authors' contributions

PL, MN, JK, and CN conceived the idea, planned and designed the study protocol. PL wrote the first draft; SF, IC, JMB, MN, JK, and CN all edited the draft and provided critical insights. All authors have approved and contributed to the final submitted manuscript.

Funding statement

This review is being supported by the Nuffield Department of Population Health (NDPH) at the University of Oxford which funds PL's doctoral research (Ref: MSD2021_119305) and the Medical Research Council (GCRF) Career Development Award (Grant Ref: MR/P022030/1)

For peer review only - http://bmjopen.bmj.com/site/about/guidelines.xhtml

which funds MN. The funders had no role in the study protocol design, review process or writing of the paper. PL had full access to all the information for the paper and had the final responsibility for the decision to submit for publication.

Competing interests' statement

The authors declare no conflict of interest.

REFERENCES

[1] Bearak J, Popinchalk A, Ganatra B, et al. Unintended pregnancy and abortion by income, region, and the legal status of abortion: estimates from a comprehensive model for 1990–2019. *Lancet Glob Heal* 2020; 8: e1152–e1161.

[2] Tsuyoshi onda SSLRGS. LK. Uneven Progress and Unequal Access. *Abort Worldw 2017 Uneven Prog Unequal Access is available online at, Visit https//www.guttmacher.org/report/abortion-worldwide-2017* 2017; 1–68.

[3] Ganatra B, Gerdts C, Rossier C, et al. Articles Global, regional, and subregional classification of abortions by safety, 2010-14: estimates from a Bayesian hierarchical model. *www.thelancet.com*; 390. Epub ahead of print 2017. DOI: 10.1016/S0140-6736(17)31794-4.

[4] Say L, Chou D, Gemmill A, et al. Articles Global causes of maternal death: a WHO systematic analysis. 2014; 323.

[5] Vlassoff M, Shearer J, Walker D, et al. *Economic Impact of Unsafe Abortion-Related Morbidity and Mortality: Evidence and Estimation Challenges*. 2008.

[6] James SL, Abate D, Abate KH, et al. Global, regional, and national incidence, prevalence, and years lived with disability for 354 Diseases and Injuries for 195 countries and territories, 1990-2017: A systematic analysis for the Global Burden of Disease Study 2017. *Lancet* 2018; 392: 1789–1858.

[7] Yokoe R, Rowe R, Choudhury SS, et al. Unsafe abortion and abortion-related death among 1.8 million women in India. *BMJ Glob Heal* 2019; 4: 1–13.

[8] Prada E, Atuyambe LM, Blades NM, et al. Incidence of Induced Abortion in Uganda, 2013: New Estimates Since 2003. Epub ahead of print 2016. DOI: 10.1371/journal.pone.0165812.

[9] Jayaweera RT, Ngui FM, Hall KS, et al. Women's experiences with unplanned pregnancy and abortion in Kenya: A qualitative study. *PLoS One* 2018; 13: e0191412.

[10] Sundari Ravindran TK, Balasubramanian P. 'Yes' to abortion but 'no' to sexual rights: The

For peer review only

For peer review only - http://bmjopen.bmj.com/site/about/guidelines.xhtml

paradoxical reality of married women in rural Tamil Nadu, India. *Reprod Health Matters* 2004; 12: 88–99.

[11] Freeman E, Coast E, Murray SF. Men's Roles in Women's Abortion Trajectories in Urban Zambia. *Int Perspect Sex Reprod Health* 2017; 43: 89–98.

[12] Engelbert Bain L, Zweekhorst MBM, Amoakoh-Coleman M, et al. To keep or not to keep? Decision making in adolescent pregnancies in Jamestown, Ghana. *PLoS One* 2019; 14: e0221789.

[13] Hajri S, Raifman S, Gerdts C, et al. 'This Is Real Misery': Experiences of Women Denied Legal Abortion in Tunisia. *PLoS One* 2015; 10: 1–16.

[14] Braam T, Hessini L. The power dynamics perpetuating unsafe abortion in Africa: a feminist perspective. *Afr J Reprod Health* 2004; 8: 43–51.

[15] Coast E, Murray SF. 'These things are dangerous': Understanding induced abortion trajectories in urban Zambia. *Soc Sci Med* 2016; 153: 201–209.

[16] Coast E, Norris AH, Moore AM, et al. Trajectories of women's abortion-related care: A conceptual framework. *Soc Sci Med* 2018; 200: 199–210.

[17] Engelbert Bain L, Amoakoh-Coleman M, Tiendrebeogo K-SST, et al. Attitudes towards abortion and decision-making capacity of pregnant adolescents: perspectives of medicine, midwifery and law students in Accra, Ghana. *Eur J Contracept Reprod Health Care* 2020; 25: 151–158.

[18] Ouedraogo R, Senderowicz L, Ngbichi C. "I wasn't ready": abortion decision-making pathways in Ouagadougou, Burkina Faso. *Int J Public Health* 2020; 65: 477–486.

[19] Puri M, Ingham R, Matthews Z. Factors Affecting Abortion Decisions among Young Couples in Nepal. *J Adolesc Heal* 2007; 40: 535–542.

[20] Rehnström Loi U, Lindgren M, Faxelid E, et al. Decision-making preceding induced abortion: A qualitative study of women's experiences in Kisumu, Kenya 11 Medical and Health Sciences 1117 Public Health and Health Services. *Reprod Health* 2018; 15: 1–12.

[21] WHO. *Medical Management of Abortion*. 2018. Epub ahead of print 2018. DOI: 10.1097/AOG.0b013e3181e8b073.

[22] Baum SE, Ramirez AM, Larrea S, et al. "It's not a seven-headed beast": abortion experience among women that received support from helplines for medication abortion in restrictive settings. *Health Care Women Int*. Epub ahead of print 2020. DOI: 10.1080/07399332.2020.1823981.

[23] Moher D, Shamseer L, Clarke M, et al. *Preferred reporting items for systematic review and meta-analysis protocols (PRISMA-P) 2015 statement*. 2015. Epub ahead of print 2015. DOI: 10.1186/2046-4053-4-1.

[24] United Nations. *The Millennium Development Goals Report*. 2015.

For peer review only - http://bmjopen.bmj.com/site/about/guidelines.xhtml

[25] United Nations. List of Millennium Development Goals , and Goal 8 Targets and Indicators Goals 1 to 7 Goal 8 : Develop a global partnership for development Targets Indicators *. 100.

[26] World Bank Group. World Bank Country and Lending Groups – World Bank Data Help Desk. *2020*, https://datahelpdesk.worldbank.org/knowledgebase/articles/906519-world-bank-country-and-lending-groups (accessed 7 January 2021).

[27] Ma LL, Wang YY, Yang ZH, et al. Methodological quality (risk of bias) assessment tools for primary and secondary medical studies: What are they and which is better? *Mil Med Res* 2020; 7: 1–11.

[28] Sterne JAC, Savović J, Page MJ, et al. RoB 2: A revised tool for assessing risk of bias in randomised trials. *BMJ* 2019; 366: 1–8.

[29] Sterne JA, Hernán MA, Reeves BC, et al. ROBINS-I: A tool for assessing risk of bias in non-randomised studies of interventions. *BMJ* 2016; 355: 4–10.

[30] Stang A. Critical evaluation of the Newcastle-Ottawa scale for the assessment of the quality of nonrandomized studies in meta-analyses. *Eur J Epidemiol* 2010; 25: 603–605.

[31] Wells G, Shea B, O'Connell D, et al. The Newcastle-Ottawa Scale (NOS) for assessing the quality if nonrandomized studies in meta-analyses. *(Available from URL http//www.ohri.ca/programs/clinical_epidemiology/oxford.asp)*. Epub ahead of print 2012. DOI: 10.2307/632432.

[32] Moola S, Munn Z, Tufanaru C, et al. Checklist for Analytical Cross Sectional Studies. *Joanna Briggs Institute Reviewer's Manual*.

[33] Moola S, Munn Z, Sears K, et al. Conducting systematic reviews of association (etiology): The Joanna Briggs Institute's approach. *Int J Evid Based Healthc* 2015; 13: 163–169.

[34] Critical Appraisal Skills Programme. CASP Qualitative Checklist. *Casp* 2018; 1.

[35] Daly J, Willis K, Small R, et al. A hierarchy of evidence for assessing qualitative health research. *J Clin Epidemiol* 2007; 60: 43–49.

[36] Barnett-Page E, Thomas J. Methods for the synthesis of qualitative research: A critical review. *BMC Med Res Methodol*; 9. Epub ahead of print 2009. DOI: 10.1186/1471-2288-9-59.

[37] Noyes J, Booth A, Moore G, et al. Synthesising quantitative and qualitative evidence to inform guidelines on complex interventions: clarifying the purposes, designs and outlining some methods. *BMJ Glob Heal* 2019; 4: e000893.

[38] Hong QN, Pluye P, Bujold M, et al. Convergent and sequential synthesis designs: implications for conducting and reporting systematic reviews of qualitative and quantitative evidence. *Syst Rev* 2017; 6: 61.

[39] Thomas J, Harden A. Methods for the thematic synthesis of qualitative research in systematic reviews. *BMC Med Res Methodol* 2008; 8: 1–10.

For peer review only

For peer review only - http://bmjopen.bmj.com/site/about/guidelines.xhtml

[40] Atkins S, Lewin S, Smith H, et al. Conducting a meta-ethnography of qualitative literature: Lessons learnt. *BMC Med Res Methodol* 2008; 8: 1–10.

[41] France EF, Uny I, Ring N, et al. A methodological systematic review of meta-ethnography conduct to articulate the complex analytical phases 11 Medical and Health Sciences 1117 Public Health and Health Services. *BMC Med Res Methodol* 2019; 19: 1–18.

[42] Carroll C, Booth A, Cooper K. A worked example of 'best fit' framework synthesis: A systematic review of views concerning the taking of some potential chemopreventive agents. *BMC Med Res Methodol*; 11. Epub ahead of print 2011. DOI: 10.1186/1471-2288-11-29.

[43] Carroll C, Booth A, Leaviss J, et al. 'best fit' framework synthesis: Refining the method. *BMC Med Res Methodol*; 13. Epub ahead of print 2013. DOI: 10.1186/1471-2288-13-37.

[44] Wan X, Wang W, Liu J, et al. Estimating the sample mean and standard deviation from the sample size, median, range and/or interquartile range. *BMC Med Res Methodol* 2014; 14: 1–13.

[45] Kimball ABBAB, Guérin A, Tsaneva M, et al. Cochrane Handbook for Systematic Reviews of Interventions Version 5.1.0 [updated March 2011]. The Cochrane Collaboration, 2011. Available from www.handbook.cochrane.org. *Br J Dermatol*.

[46] Noyes J, Booth A, Flemming K, et al. Cochrane Qualitative and Implementation Methods Group guidance series—paper 3: methods for assessing methodological limitations, data extraction and synthesis, and confidence in synthesized qualitative findings. *J Clin Epidemiol* 2018; 97: 49–58.

[47] Lewin S, Booth A, Glenton C, et al. Applying GRADE-CERQual to qualitative evidence synthesis findings: Introduction to the series. *Implement Sci* 2018; 13: 1–10.

[48] BMJ. What is GRADE? *BMJ Clin Evid*.

Word count: 2650

For peer review only - http://bmjopen.bmj.com/site/about/guidelines.xhtml

For peer review only

[i]PRISMA-P (Preferred Reporting Items for Systematic review and Meta-Analysis Protocols) 2015 checklist: recommended items to address in a systematic review protocol*

Section and topic	Item No	Checklist item	Self-Evaluation
**ADMINISTRATIVE INFORMATION**			
Title:			
Identification	1a	Identify the report as a protocol of a systematic review	**YES, identified (title page)**
Update	1b	If the protocol is for an update of a previous systematic review, identify as such	**Not applicable**
Registration	2	If registered, provide the name of the registry (such as PROSPERO) and registration number	**CRD42021224719 (title page, page 5)**
Authors:			
Contact	3a	Provide name, institutional affiliation, e-mail address of all protocol authors; provide physical mailing address of corresponding author	**YES, it is provided (Title page)**
Contributions	3b	Describe contributions of protocol authors and identify the guarantor of the review	**YES, this is provided (page 12)**
Amendments	4	If the protocol represents an amendment of a previously completed or published protocol, identify as such and list changes; otherwise, state plan for documenting important protocol amendments	**Not applicable**
Support:			
Sources	5a	Indicate sources of financial or other support for the review	**Yes (page 12)**
Sponsor	5b	Provide name for the review funder and/or sponsor	**Yes (page 12-13)**
Role of sponsor or funder	5c	Describe roles of funder(s), sponsor(s), and/or institution(s), if any, in developing the protocol	**Yes (page 12-13)**
**INTRODUCTION**			
Rationale	6	Describe the rationale for the review in the context of what is already known	**Yes (page 4)**
Objectives	7	Provide an explicit statement of the question(s) the review will address with reference to participants, interventions, comparators, and outcomes (PICO)	**Yes (page 4)**
**METHODS**			
Eligibility criteria	8	Specify the study characteristics (such as PICO, study design, setting, time frame) and report characteristics (such as years considered, language, publication status) to be used as criteria for eligibility for the review	**Yes (page 6-7)**
Information sources	9	Describe all intended information sources (such as electronic databases, contact with study authors, trial registers or other grey literature sources) with planned dates of coverage	**Yes (page 5)**
Search strategy	10	Present draft of search strategy to be used for at least one electronic database, including planned limits, such that it could be repeated	**Yes (supplementary file 2)**

For peer review only

Study records:			
Data management	11a	Describe the mechanism(s) that will be used to manage records and data throughout the review	**Yes (pages 7-8)**
Selection process	11b	State the process that will be used for selecting studies (such as two independent reviewers) through each phase of the review (that is, screening, eligibility and inclusion in meta-analysis)	**Yes (pages 7-10)**
Data collection process	11c	Describe planned method of extracting data from reports (such as piloting forms, done independently, in duplicate), any processes for obtaining and confirming data from investigators	**Yes (pages 7-9)**
Data items	12	List and define all variables for which data will be sought (such as PICO items, funding sources), any pre-planned data assumptions and simplifications	**Yes (pages 8-9)**
Outcomes and prioritization	13	List and define all outcomes for which data will be sought, including prioritization of main and additional outcomes, with rationale	**Yes (pages 8-9)**
Risk of bias in individual studies	14	Describe anticipated methods for assessing risk of bias of individual studies, including whether this will be done at the outcome or study level, or both; state how this information will be used in data synthesis	**Yes (pages 9-10)**
Data synthesis	15a	Describe criteria under which study data will be quantitatively synthesised	**Yes (page 11)**
	15b	If data are appropriate for quantitative synthesis, describe planned summary measures, methods of handling data and methods of combining data from studies, including any planned exploration of consistency (such as I^2, Kendall's τ)	**Yes (page 11)**
	15c	Describe any proposed additional analyses (such as sensitivity or subgroup analyses, meta-regression)	**Yes (page 11)**
	15d	If quantitative synthesis is not appropriate, describe the type of summary planned	**Yes (page 10)**
Meta-bias(es)	16	Specify any planned assessment of meta-bias(es) (such as publication bias across studies, selective reporting within studies)	**Yes (page 11)**
Confidence in cumulative evidence	17	Describe how the strength of the body of evidence will be assessed (such as GRADE)	**Yes (page 11)**

*** It is strongly recommended that this checklist be read in conjunction with the PRISMA-P Explanation and Elaboration (cite when available) for important clarification on the items. Amendments to a review protocol should be tracked and dated. The copyright for PRISMA-P (including checklist) is held by the PRISMA-P Group and is distributed under a Creative Commons Attribution Licence 4.0.**

From: Shamseer L, Moher D, Clarke M, Ghersi D, Liberati A, Petticrew M, Shekelle P, Stewart L, PRISMA-P Group. Preferred reporting items for systematic review and meta-analysis protocols (PRISMA-P) 2015: elaboration and explanation. BMJ. 2015 Jan 2;349(jan02 1):g7647.

i

For peer review only

Abortion decision-making trajectories and determinants in low- and middle-income countries: a protocol for mixed methods systematic review and meta-analysis
Preliminary sample search for Ovid Medline: Search date 28/12/2020

#	Search	Results
1	(afghanistan or albania or algeria or american samoa or angola or "antigua and barbuda" or antigua or barbuda or argentina or armenia or armenian or aruba or azerbaijan or bahrain or bangladesh or barbados or republic of belarus or belarus or byelarus or belorussia or byelorussian or belize or british honduras or benin or dahomey or bhutan or bolivia or "bosnia and herzegovina" or bosnia or herzegovina or botswana or bechuanaland or brazil or brasil or bulgaria or burkina faso or burkina fasso or upper volta or burundi or urundi or cabo verde or cape verde or cambodia or kampuchea or khmer republic or cameroon or cameron or cameroun or central african republic or ubangi shari or chad or chile or china or colombia or comoros or comoro islands or iles comores or mayotte or democratic republic of the congo or democratic republic congo or congo or zaire or costa rica or "cote d'ivoire" or "cote d' ivoire" or cote divoire or cote d ivoire or ivory coast or croatia or cuba or cyprus or czech republic or czechoslovakia or djibouti or french somaliland or dominica or dominican republic or ecuador or egypt or united arab republic or el salvador or equatorial guinea or spanish guinea or eritrea or estonia or eswatini or swaziland or ethiopia or fiji or gabon or gabonese republic or gambia or "georgia (republic)" or georgian or ghana or gold coast or gibraltar or greece or grenada or guam or guatemala or guinea or guinea bissau or guyana or british guiana or haiti or hispaniola or honduras or hungary or india or indonesia or timor or iran or iraq or isle of man or jamaica or jordan or kazakhstan or kazakh or kenya or "democratic people's republic of korea" or republic of korea or north korea or south korea or korea or kosovo or kyrgyzstan or kirghizia or kirgizstan or kyrgyz republic or kirghiz or laos or lao pdr or "lao people's democratic republic" or latvia or lebanon or lebanese republic or lesotho or basutoland or liberia or libya or libyan arab jamahiriya or lithuania or macau or macao or republic of north macedonia or macedonia or madagascar or malagasy republic or malawi or nyasaland or malaysia or malay federation or malaya federation or maldives or indian ocean islands or indian ocean or mali or malta or micronesia or federated states of micronesia or kiribati or marshall islands or nauru or northern mariana islands or palau or tuvalu or mauritania or mauritius or mexico or moldova or moldovian or mongolia or montenegro or morocco or ifni or mozambique or portuguese east africa or myanmar or burma or namibia or nepal or netherlands antilles or nicaragua or niger or nigeria or oman or muscat or pakistan or panama or papua new guinea or new guinea or paraguay or peru or philippines or philipines or phillipines or phillippines or poland or "polish people's republic" or portugal or portuguese republic or puerto rico or romania or russia or russian federation or ussr or soviet union or union of soviet socialist republics or rwanda or ruanda or samoa or pacific islands or polynesia or samoan islands or navigator island or navigator islands or "sao tome and principe" or saudi arabia or senegal or serbia or seychelles or sierra leone or slovakia or slovak republic or slovenia or melanesia or solomon island or solomon islands or norfolk island or norfolk islands or somalia or south africa or south sudan or sri lanka or ceylon or "saint kitts and nevis" or "st. kitts and nevis" or saint lucia or "st. lucia" or "saint vincent and the grenadines" or	1981780

For peer review only

	saint vincent or "st. vincent" or grenadines or sudan or suriname or surinam or dutch guiana or netherlands guiana or syria or syrian arab republic or tajikistan or tadjikistan or tadzhikistan or tadzhik or tanzania or tanganyika or thailand or siam or timor leste or east timor or togo or togolese republic or tonga or "trinidad and tobago" or trinidad or tobago or tunisia or turkey or turkmenistan or turkmen or uganda or ukraine or uruguay or uzbekistan or uzbek or vanuatu or new hebrides or venezuela or vietnam or viet nam or middle east or west bank or gaza or palestine or yemen or yugoslavia or zambia or zimbabwe or northern rhodesia or global south or africa south of the sahara or sub-saharan africa or subsaharan africa or africa, central or central africa or africa, northern or north africa or northern africa or magreb or maghrib or sahara or africa, southern or southern africa or africa, eastern or east africa or eastern africa or africa, western or west africa or western africa or west indies or indian ocean islands or caribbean or central america or latin america or "south and central america" or south america or asia, central or central asia or asia, northern or north asia or northern asia or asia, southeastern or southeastern asia or south eastern asia or southeast asia or south east asia or asia, western or western asia or europe, eastern or east europe or eastern europe or developing country or developing countries or developing nation? or developing population? or developing world or less developed countr* or less developed nation? or less developed population? or less developed world or lesser developed countr* or lesser developed nation? or lesser developed population? or lesser developed world or under developed countr* or under developed nation? or under developed population? or under developed world or underdeveloped countr* or underdeveloped nation? or underdeveloped population? or underdeveloped world or middle income countr* or middle income nation? or middle income population? or low income countr* or low income nation? or low income population? or lower income countr* or lower income nation? or lower income population? or underserved countr* or underserved nation? or underserved population? or underserved world or under served countr* or under served nation? or under served population? or under served world or deprived countr* or deprived nation? or deprived population? or deprived world or poor countr* or poor nation? or poor population? or poor world or poorer countr* or poorer nation? or poorer population? or poorer world or developing econom* or less developed econom* or lesser developed econom* or under developed econom* or underdeveloped econom* or middle income econom* or low income econom* or lower income econom* or low gdp or low gnp or low gross domestic or low gross national or lower gdp or lower gnp or lower gross domestic or lower gross national or lmic or lmics or third world or lami countr* or transitional countr* or emerging economies or emerging nation?).ti,ab,sh,kw.	
2	exp Abortion, Induced/	40425
3	abortion.mp.	88886
4	pregnancy termination.mp.	2413
5	2 or 3 or 4	90396
6	decision making.mp.	222374
7	exp Decision Making/	206294
8	6 or 7	327808

For peer review only

9	1 and 5 and 8	496
10	limit 9 to (humans and yr="2000 -Current")	321

For peer review only
